# Photophysics and photochemistry of NIR absorbers derived from cyanines: key to new technologies based on chemistry 4.0

**DOI:** 10.3762/bjoc.16.40

**Published:** 2020-03-18

**Authors:** Bernd Strehmel, Christian Schmitz, Ceren Kütahya, Yulian Pang, Anke Drewitz, Heinz Mustroph

**Affiliations:** 1 Niederrhein University of Applied Sciences, Department of Chemistry and Institute for Coatings Surface Chemistry, Adlerstr. 1, D-47798 Krefeld, Germany; 2 GMBU e.V., Felsbachstraße 7, D-07745 Jena, Germany; 3 formerly at FEW Chemicals GmbH, Technikumstraße 1, D-06766 Bitterfeld-Wolfen, Germany

**Keywords:** chemistry 4.0, cyanine, near infrared, photopolymer, polymer synthesis, sensitizer

## Abstract

Cyanines derived from heptamethines were mainly discussed regarding their functionalization to broaden the solubility in different surroundings exhibiting either hydrophilic or hydrophobic properties and to tailor made the Δ*G*_et_ photopysical properties with respect to absorption and fluorescence. Electrochemical properties were additionally considered for some selected examples. The cyanines chosen comprised as end groups either indolenine, benzo[*e*]- or benzo[*cd*]indolium pattern, which facilitated to shift the absorption between 750–1000 nm. This enabled their use in applications with light sources emitting in the near-infrared (NIR) region selected from high power LEDs or lasers with line-shaped focus. The absorbers considered were discussed regarding their function as sensitizer for applications related to Chemistry 4.0 standards. These were mainly photopolymer coatings, which can be found for applications in the graphic industry or to protect selected substrates. The huge release of heat on demand upon turning ON or OFF the NIR light source enables them for photothermal treatment in processes requesting heat to initiate either chemical (activated reactions) or physical (melting, evaporation) events.

## Introduction

Cyanines have received in the class of polymethines big attention within the last hundred years [1–4]. Their substitution pattern easily facilitates shift of the absorption from the ultra violet part into the near infrared region while they exhibit huge extinction coefficients [3]. The connection of two nitrogens with an odd number of methine groups results in a positive charge of the final molecule if the cyanine comprises no additional substituent exhibiting a negative charge at the molecular skeleton. Furthermore, it requires to combine this cation with appropriate anions to achieve a good compatibility with the surrounding matrix. Compatibility in these examples preferably refers to the solubility in the surrounding matrix, while undesirable events such as aggregation are of minor importance. In the worst case, it can also result in crystallization of the cyanine in the matrix under certain storage conditions such as high humidity and/or elevated temperatures available in some geographic areas where these materials have been used in practice. Such anions can be either bis(trifluoromethyl)sulfonylimides [5], aluminates [6] or sulfonates comprising long alkyl chains [5]. Alternatively, a barbiturate group positioned at the *meso*-position of the cyanine can also sometimes lead to unexpectedly high solubility in aprotic polar surroundings [5]. Nevertheless, a variation between the cation of the cyanine with distinct anions may move such materials to applications where either an aqueous surrounding being available in biological applications [7–9] or a dipolar aprotic matrix takes the function of the matrix with absorber embedded. Typically, digital imaging in Computer to Plate technology (CtP) [10–14], curing of liquid coatings [5,15], powder coatings [16–17], laser drying [18–20], laser welding [21–25], or laser marking of plastics [26–31] represent some practical examples. Nevertheless, cyanines have held a long history regarding their practical use, which started to use such materials as sensitizers in silver halide photography [32–34] until the point when electronic media took their place in imaging sciences to save pictures. Many fundamental knowledge was grown up in this period as shown for example by several reviews [35–40]. Particularly, the use of model systems and the knowledge obtained by exploration systems in silver halide photography [32–34,41–45] also helped to understand the function and formation *H* and *J*-aggregates [46–49] being aligned parallel or in series, respectively. The capability of cyanines to form *J*-aggregates exhibiting a huge bathochromic shift, which was reported for the first time by Scheibe [50] and Jelley in 1937 [51], enabled spectral sensitization of AgX photography over a long spectral range [41–45]. Though AgX photography has not been alive at large scale, research pursued in this field helped to develop theories regarding the design of absorbers/sensitizers and their function in other industrial applications.

Cyanines possess an odd number 2*n* + 3 of π-centers and 2*n* + 4 π-electrons [1]. The variable *n* represents the number of vinylene groups in the methine chain. The absorption of cyanines can be tailor made by extension of the methine chain by two methine groups of the vinylene moiety resulting in a bathochromic shift of the absorption of about 100 nm [3]. Substitution of the terminal groups comprising by either substituted indolium, benzo[*e*]indolium or benzo[*c,d*]indolium patterns helps to finetune the absorption wavelength with respect to the light source used. Furthermore, different substituents at the *meso*-position comprising either electron-donating or electron-withdrawing substituents complement the design of tailor made absorption [52–54]. The fact that many investigations were pursued in the visible range assigned these materials in general as dyes, which should not be correct. The term dye relates to the visible part covering the range between 400–700 nm considering the longest absorption maximum [55]. The use of the term absorber fits better as a general assignment that also relates to the absorption range below and above this range. Furthermore, one can also name the function of a cyanine in a given application which can be either a sensitizer, initiator, staining, activator or a filter material just to name a few possible examples. Unfortunately, literature often does not clearly distinguish between their functionality.

Besides cyanines, there were made huge efforts to synthesize alternatives such as rylenes [56] or conjugated polymers [57] with the focus to receive materials covering a broad absorption range up to the near infrared (NIR) part. Nevertheless, they have not reached the necessary practical use as cyanines explainable by the modest solubility in many industrial matrix materials used for different applications. These facts advantageously demonstrate the benefits of cyanines where tailor-made synthesis of the unsaturated cation and the selection of an appropriate anion facilitated sufficient solubility with many matrix materials [5–6]. In comparison, cyanines depict from this point of view outstanding properties since the aforementioned benefits enabled their use in photopolymerization to pattern light sensitive coatings [11–14], to cure liquid coatings [15], and powder coatings [16–17] or to initiate either a chemical and/or thermal curing process [58].

Although some photophysical studies disclose the absorption and emission properties [13,59–62], comparison of available data may lead sometimes to contradictory conclusions. This article first summarizes possibilities for synthesis of different patterns of cyanines exhibiting indolenine and benzindolenine moiety at the ends of the methine chain while the cyanine pattern also changed at the center of the molecule. A summary of determined and almost published photophysical and electrochemical data should complement available data available [3,13–14,59–62] to provide a brief idea regarding their use in applications based on sensitized photoinduced electron transfer. This can be NIR-sensitized photopolymerization resulting in formation of initiating radicals and conjugate acid [5–6,13–15,63–64]. Recently, the use of NIR-LEDs exhibiting high excitation intensity brought more light in this field and helped to understand the function of existing intrinsic barrier in such systems comprising cationic cyanines in photoinduced electron transfer systems [65]. Furthermore, the huge amount of heat released by nonradiative deactivation of the excited state brings up to use them as “molecular oven/furnace” in heat-based physical and/or chemical processes. There, they can generate heat in an ON/OFF procedure on demand [58].

Many examples of cyanines focus on the use of absorption in the NIR [64]. Among of the disclosed applications, photopolymerization depicts one representative method where the absorber uptakes the function of a sensitizer to generate initiating radicals and conjugate acid [13–17,58,65]. The fact that these systems work upon turning ON/OFF cycles facilitates their use in Industry 4.0 related applications where printing represents one feasible application [66]. Data collection [67] in combination with self-leaning software related to artificial intelligence (A.I.) drives their use in applications with more efficiency in large workflows. Such tools can recognize failures in industrial processes just by collection of data in a big database where data analysis of global production may solve issues related to the materials.

## Review

### Synthesis of cyanines absorbing in the NIR

**Scheme 1:**
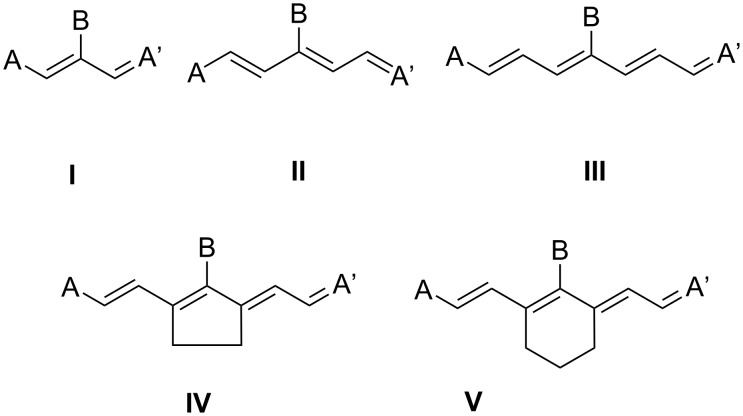
Structural patterns of several symmetric cyanines relating to trimethines (**I**), pentamethines (**II**), and heptamethines (**III**–**V**). While **I**-**III** exhibit an open methine chain, functionalization by bridged moieties results in either a planar (**IV**) or non-planar (**V**) structure. The terminal group A/A’ belongs to indolium, benzo[*e*]indolium, and benzo[*c,d*]indolium (Scheme 2). **I**–**V** comprise a further substituent in the *meso*-position.

**Scheme 2:**
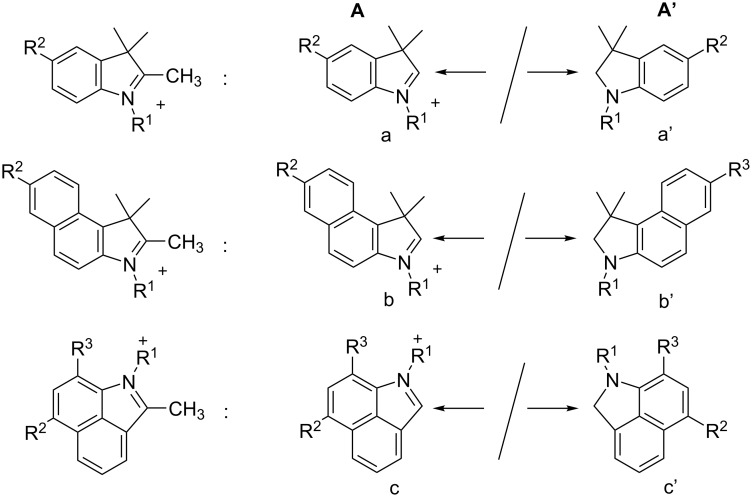
1-Substituted 2,3,3-trimethylindolium-, 2,3,3-benzo[*e*]indolium-, and 2,3,3-benzo[*c,d*]indolium salts lead to the patterns a/a’, b/b’ and c/c’ of the terminal substituent **A**, respectively.

Cyanines comprising the patterns **I**–**V** (Scheme 1) have received much importance as NIR absorbers applied in different technologies [1,8–9,11–20,32–34]. They exhibit a flexible chain (**I–III**) or bridged chain (**IV**, **V**) where the cyclopentene pattern keeps the system planar while the introduction of the non-planar cyclohexene moiety results in improvement of compatibility of the surrounding matrix [5]. Indolium, benzo[*e*]indolium, and benzo[*c,d*]indolium (Scheme 2) represent the most widely used substitution patterns of NIR absorbers. Their structures formed can be easily build together following the procedures disclosed [2] while patent procedures also worked well [68–69].

These structures depict only one valence pattern while the positive charge typically distributes over the entire conjugated system. There are much more valence patterns possible to draw. For a sake of simplicity, we operate with one feasible structure. The availability of these precursors bases on easy pursuable chemical reactions resulting in huge libraries of cyanines exhibiting different substitution patterns.

Thus, **I**–**V** belong to symmetric cyanines whose synthesis has been known for a long time [2]. This also explains why there exist plenty of procedures facilitating the synthesis with high yields and acceptable purity of the crude reaction products. These efforts helped to enable such materials to technologies requesting a higher demand on materials; that is for example the graphic industry where these absorbers have been successfully worked in Computer to Plate (CtP) applications for more than 25 years [11–14]. Nowadays, manufacturing of symmetric cyanines approaches up to several tons worldwide/year in the aforementioned applications. On the other hand, cyanines exhibiting asymmetric structural patterns have also reached practical fields but this relates more or less to medical and biological uses [7–9] with rather low but exclusive quantities. These requests enforced to introduce special functional groups facilitating their use as marker in the aforementioned fields. Nevertheless, applications relating to biology or medicine demand less economic pressure because the use of these compounds typically resides at the milligram or gram-scale.

The methine chain can be either open as in **I**–**III** facilitating a certain flexibility in the main chain or bridged resulting in either a five-membered (**IV**) or six-membered (**V**) moiety. **IV** exhibits almost a planar structure while the more flexible 6-membered unit in the center of **V** results in a non-planar geometry [63]. Such a geometry affects compatibility with the surrounding matrix. Thus, aggregation phenomena dominate in the case of **IV**. On the other hand, absorbers related to **V** often exhibit a much better solubility in organic surroundings such as multi-functional acrylates [5]. This can be seen as a huge challenge – obtaining a sufficient solubility of such ionic materials in a surrounding being aprotic polar and highly viscous. Many absorbers failed to meet these requirements in practice although their fundamental research pursued in highly diluted solutions showed promising directions [5]

The substitution of either **I**, **II**, **III**, **IV** or **V** with distinct terminal groups **A**/**A’** results in cyanines exhibiting either a cationic, zwitterionic or anionic pattern. Their solubility covers a wide range including aprotic polar organic solvents or also water. The latter requests to introduce functional groups promoting their water solubility; that is the sulfobutyl group in the case of R^1^ or -SO_3_^−^ in the case of either R^2^ or R^3^. Water soluble cyanines could feasibly favor physical drying of aqueous dispersions upon irradiation with a NIR-LED [70]. In addition, chemical drying of coatings requires the use of strong anions promoting the solubility in organic surroundings. Representative anions in the case of cationic absorbers relate to tosylate [5], [*n*-C_12_H_25_-Ph-SO_3_^−^] [5], FAP ([(C_2_F_5_)_3_PF_3_]^−^)[71], NTf_2_ ([(CF_3_SO_2_)_2_N]^−^) [5] or aluminates ([Al(*t*-C_4_F_9_O)_4_]^−^) [6] – just to count a few possible examples. Thus, replacement of the counter ion can sometimes unexpectedly improve the solubility as shown for some cationic absorbers comprising either the NTf_2_-anion [5] or the aforementioned aluminate anion [6] where the solubility of the respective absorber can approach 10–30 g/L in multi-functional acrylic esters [5]. In addition, small structural changes of the alkyl substituent R^1^ even caused big differences regarding the solubility in the surrounding matrix. This field, namely driving of absorber solubility with the surrounding matrix by the structural pattern of both the cyanine and the respective counter ion, has not been well understood yet. It depicts rather semi-empiric approaches nowadays. More theoretical works would definitely bring more light in this field while the vide supra mentioned applications would definitively benefit from such studies.

**Scheme 3:**
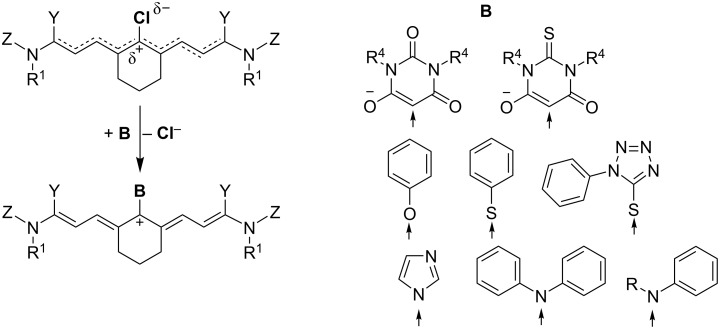
Substitution of the chlorine substituent at the *meso*-position by a stronger nucleophilic moiety **B** [68].

**Scheme 4:**
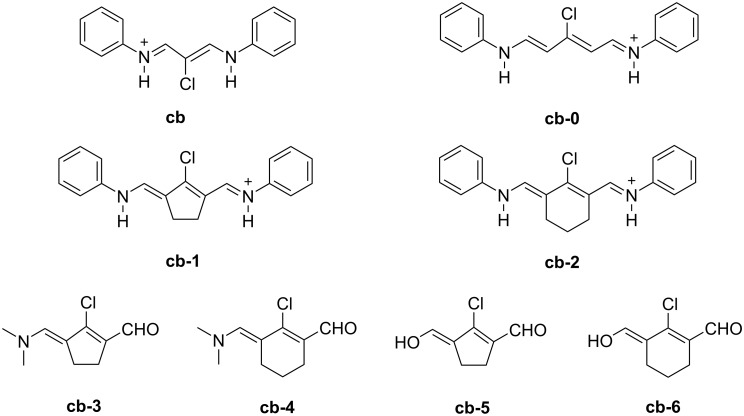
Structure of alternative chain builders for synthesis of heptamethines.

Synthesis of **I**–**V** proceeds according to the reactions shown in Scheme 3. It uses the indolium derivatives shown in Scheme 2 as starting materials. They can react either with ortho esters, tris-halogenated precursors or diphenylformamidines resulting in trimethine cyanines [2]. The latter reference depicts much more reagents resulting in the desired trimethine patterns. Moreover, synthesis of pentamethine cyanines **II** follows a route requesting **cb** (Scheme 4) as precursor. There serve some aldehydes as source to synthesize this precursor by reaction with aniline. Similar reaction philosophy also follows synthesis of **cb-0** (Scheme 4) to synthesize open heptamethines **III**. Particularly, an additional substituent at the *meso*-position introduces more freedoms to functionalize such materials to obtain an absorption being tailor made with respect to the light source applied. This can be a semiconductor laser [14–16,18–20] or high-power NIR-LED [65] with emission between 800–1100 nm. Such findings enforce activities to make absorbers exhibiting internal barriers in photoinduced electron transfer reactions [72] resulting in a certain white light stability under ambient light conditions. Such properties can be seen as a big benefit from a practical point of view in comparison with UV-sensitive materials.

Synthesis of the bridged heptamethines (**IV** and **V**) follows the general route shown in Scheme 3 [2,68–69]. Scheme 4 discloses the use of the chain builders **cb** and **cb0-cb-6** [68–69]. The materials **cb-1** and **cb-2** were available by reaction of the respective cyclic ketone with POCl_3_ and DMF in CH_2_Cl_2_ in the case of the amino substituted derivatives in high yields. This reference also describes the synthesis of the chain builders **cb-3** and **cb-4**. It occurred by a similar procedure. Synthesis of **cb5** and **cb6** was already reported [68].

The heptamethine pattern also facilitated replacement of the substituent at the *meso*-position. Scheme 3 shows the activation of the carbon at this position by the chlorine substituent. This explains why functionalization of cyanine pattern by either electron withdrawing or electron donating substituents easily proceeds. It also enabled the introduction of reactive groups such as -N=C=S facilitating them to function as marker in biological/imaging applications [9]. This reaction occurred with good yields and extended the use of such NIR absorbers in more fields [1–2,7–9]. In general, this functionalization resulted in changes of absorption maxima and electrochemical potentials appearing sometimes with rather low quantities, see Table 1 for explanation of the absorbers and Table 2 for the data vide infra.

These synthetic efforts resulted in many distinct absorbers whose absorption and electrochemical properties differ. It facilitates their use with modern light sources such LEDs and lasers with emission in the NIR between 800–1100 nm. A purposeful use requires a more detailed understanding of the photochemistry occurring in such compounds.

## Photophysics and photochemistry

### General aspects

**Figure 1:**
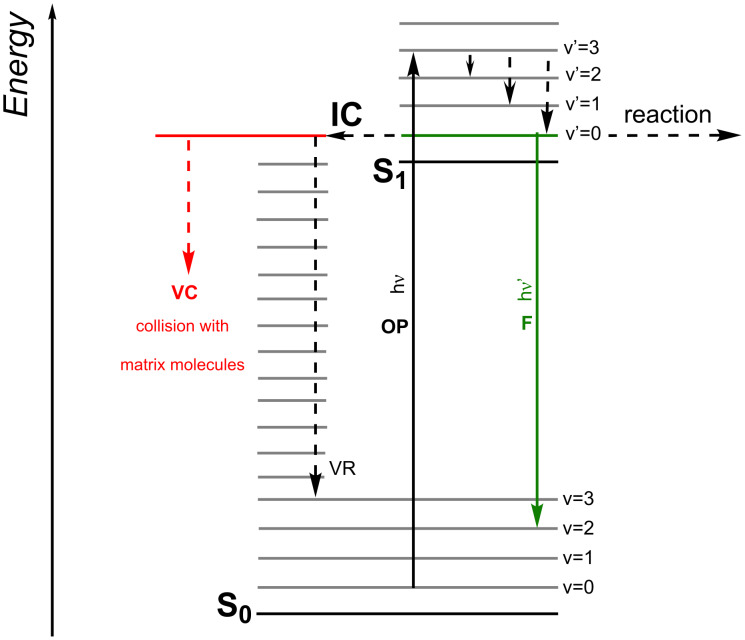
Simplified process chart of photophysical processes occurring in NIR absorbers.

Figure 1 depicts photochemistry and photophysics of NIR absorbers from the simplest point of view. Excitation based on one-photon excitation (OP) results in population of higher vibrational levels of the first excited singlet state (S_1_), which fast relax by vibrational relaxation into the lowest vibrational level of the S_1_. The excited state possesses several competitive reaction pathways for deactivation. This is a photochemical reaction such as photoinduced electron transfer serving as source to generate reactive intermediates such as initiating radicals or conjugate acid [5]. Photophysical events of S_1_ occurring after one-photon excitation (OP) include internal conversion (IC), fast vibrational relaxation (VR) from higher vibrational modes of the S_1_ (v’ = 2, v’ = 1) into its lowest vibrational mode (v’ = 0), vibrational relaxation from lowest vibrational mode of the S_1_ into higher vibrational modes (for example v = 2) of the ground state (S_0_), intermolecular energy transfer from vibrationally hot molecules to their cold surrounding (vibrational cooling = VC) [73–74], and fluorescence (F) [75–76]. Most likely, nonradiative deactivation of such a hot molecule could be the transfer of its energy by collision with matrix molecules to the surrounding (VC) occurring from higher vibrational modes of the ground state formed by IC. Photochemical reactions typically occur from the lowest vibrational level (v’ = 0) of the S_1_. This figure does not contain an intersystem crossing route resulting in triplet states because this does not play any role considering the absorbers explored in this study. It was omitted because from our best knowledge there were no triplet reactions reported for heptamethines. In general, vibrational cooling may be seen as the main contributing to heat release (Δ*T*) into the surrounding matrix caused by collision with matrix molecules. Although deactivation by vibrational coupling competitively occurs as well, this reaction should play only a minor function in the deactivation scheme. There exists too much excessive energy, which would destroy the NIR absorber if the deactivation would mainly proceed by vibrational deactivation within the molecule. Therefore, transfer of the excessive energy by collision with matrix molecules appears as the most likely alternative which does not exclude the aforementioned vibrational deactivation within the molecule. The heat generated is large enough to initiate physical processes such as melting of powder coatings [16] or activation of thermal reaction such as blocked isocyanates [58]. It can exceed, depending on excitation intensity, several 100 °C. Particularly, embedded pigments with higher heat capacity can drive such systems to temperatures greater than 350 °C [77].

Nonradiative deactivation represents the main route of NIR absorbers to transfer the S_1_ to the S_0_. Its efficiency is higher than 85% [14] exhibiting an absorption between 750–850 nm, see also Table 2 vide infra. The non-radiative deactivation of absorbers with an absorption above 900 nm occurs almost quantitatively. This can be seen as one big feature of NIR absorbers; that is the function of a “molecular oven/furnace” whose operation bases on a photonic process, which can be easily turned ON or OFF by operation of the light source. Medical applications with focus on cancer therapy named this phenomenon photothermal treatment [78–80] In addition, heat generation on demand also explains the function of laser drying developed for the graphic industry [11–14] or laser welding [21–25] where the release of heat by a photonic event responsibly patterns a positive image on lithographic materials [11–14]. Moreover, traditional processes based on furnace techniques have been widely used in industry but their uneconomic efficiency has called a demand for more energy efficient drying technologies [18–20,54]. This can be physical and/or chemical drying of coatings. Particularly, photoinduced electron transfer enables chemical drying. For this purpose, systems based on photoinduced electron transfer with internal barrier facilitate their practical use in industrial systems. The thermal energy released helps to overcome the internal barrier of the system.

Equations 1–4 summarize these events discussed above whose rate constants quantify the individual processes. They occur from the S_1_ while no triplet states have been reported yet for the cyanines shown in Table 1. A modification of these patterns with more heavy atoms would definitively drive these into the triplet state and brings benefits known from UV systems to NIR systems either. This can be the generation of singlet oxygen and would enable the use of NIR absorbers in photodynamic therapy. Another benefit can be seen to increase the efficiency of photoinduced electron transfer needed in imaging applications. This would definitively decrease the efficiency of electron back transfer. Nowadays, typical electron acceptors (AC) applied in such systems related to either iodonium salts or triazines also possess a high capability of electron back transfer resulting in a decrease of the overall efficiency. **PA** relates to a photoactive compound, which functions in the examples as sensitizer **Sens** vide infra.

[1] PA→hν(PA)1*

[2] (PA)1*→P1A+hν'

[3] (PA)1*→P1A+ΔT

[4] (PA)1*+AC→PA+⋅+AC−⋅

In practice, addition of an electron-donating compound (DN) often reduces tendency of electron back transfer in such singlet systems as shown in Equation 5 because the photo-oxidized sensitizer formed in Equation 4 reacts with DN resulting in back formation of **PA** while a new reaction intermediate DN^+^ is formed, Equation 6. This fast decomposes resulting in generation of reactive intermediates and makes the system irreversible.

[5] $$PA^{+\cdot }+AC^{-\cdot }\overset{}{\to }PA+AC$$

[6] $$PA^{+\cdot }+DN\overset{}{\to }PA+DN^{+\cdot }$$

The oxidized species (PA^+^) also competitively decomposes resulting in lower molecular weight products caused by cleavage of the methine chain and conjugate acid, see Equation 7 [5,63]. The latter can initiate ring opening polymerization of aziridines [5] and oxiranes [63]. We also prefer to use the term conjugate acid instead of proton. The use of the latter widely distributes in literature but is wrong because protons do not exist alone and data related to pH-values relate only to aqueous solutions. Ring opening by rhodamine B lactone quantitatively probed formation conjugate acid by formation of rhodamine B [5]. We interpret the term conjugate acid as a species in which the matrix binds H^+^ released by PA^+^.

[7] $$PA^{+\cdot }\overset{}{\to }\text{products + conjugate acid}$$

Table 2 summarizes photophysical and electrochemical data of several dozens of absorbers. Some of the data may serve as reference for future studies since the availability of such data could be seen as more or less rare. The benzo[*c,d*]indolium group (compounds **1**–**11**) offers the most interesting features regarding spectral variation covering a range between 750–1000 nm considering these examples. Structures 1, 2, and 5, which fit into the structural group of **I**, **II** and **III**, respectively. They demonstrate the expected absorption shift of about 100 nm by extension of the methine chain by one (CH=CH) unit. The fluorescence quantum yield of **1** and **2** was low while the extinction coefficient resided around 2 × 10^5^ M^−1^ cm^−1^. The anions used were mostly derived from BF_4_^−^, PF_6_^−^, NFT_2_^−^, and *n*-C_12_H_25_-Ph-SO_3_^−^. This helps to improve the solubility in different surroundings and brings them to practice. Particularly, absorbers comprising the C_12_H_25_-Ph-SO_3_^−^ anion for laser drying in offset printing depict one example [54]. For comparison, indolium (structure a/a’) and benzo[*e*]indolium end groups required more methine groups to obtain a comparable absorption in the NIR (compare structures **12**–**87**). The planar c/c’-pattern helped to drive the desired absorption easier in the NIR. Nevertheless, applications using such absorbers in a sensitized oxidation mechanism benefit from their use. Such photoreactions typically result in cleavage of the methine chain. Nevertheless, the obtained photoproducts should not exhibit such a strong brownish color as observed in photochemical studies to explore the heptamethines **48** [63]. Nowadays, this arises as a big challenge to use such absorbers in applications where the color formed can be seen as an issue.

**Table 1:** Summary of structure elements of absorbers related to the structural patterns **I**–**V**. Table 2 summarizes their photophysical data. FEW Chemicals GmbH provided samples for the investigations.

#	group^a^	A/A’ ^b^	B^c^	R^1^	R^2^	R^3^	counter ion^c^

**1**	**I**	c/c’	H	C_4_H_9_	H	–	BF_4_^−^
**2**	**II**	c/c’	H	C_4_H_9_	H	–	BF_4_^−^
**3**	**II**	c/c’	H	C_4_H_9_	OC_4_H_9_	–	BF_4_^−^
**4**	**II**	c/c’	H	*t*-C_4_H_9_	*t-*BuPS^c^	*t*-BuPS^c^	NTf_2_^−^
**5**	**III**	c/c’	H	C_4_H_9_	H	H	PF_6_^−^
**6**	**IV**	c/c’	(Ph)_2_N-	C_4_H_9_	H	H	BF_4_^−^
**7**	**IV**	c/c’	Ph-	C_4_H_9_	H	H	BF_4_^−^
**8**	**IV**	c/c’	Ph-	C_4_H_9_	H	H	DoPhSO_3_^−^
**9**	**V**	c/c’	Ph-	C_4_H_9_	H	H	BF_4_^−^
**10**	**V**	c/c’	Ph-	C_4_H_9_	H	H	DoPhSO_3_^−^
**11**	**V**	c/c’	Cl	C_4_H_9_	H	H	BF_4_^−^
**12**	**III**	a/a’	H	C_4_H_9_	H	–	PF_6_^−^
**13**	**III**	a/a’	H	(CH_2_)_4_SO_3_^−^	SO_3_^−^	–	3 K^+^
**14**	**V**	a/a’	Ph-O-	C_4_H_9_	H	–	PF_6_^−^
**15**	**V**	a/a’	Ph-O-	(CH_2_)_4_SO_3_^−^	H	–	Na^+^
**16**	**III**	a/a’	H	CH_3_	H	–	ClO_4_^−^
**17**	**IV**	a/a’	N(CH_3_)_2_	C_4_H_9_	H	–	PF_6_^−^
**18**	**V**	a/a’	Ph-	C_4_H_9_	H	–	PF_6_^−^
**19**	**V**	a/a’	Ph-	(CH_2_)_4_SO_3_^−^	SO_3_^−^	–	3 Na^+^
**20**	**V**	a/a’	Ph-	(CH_2_)_4_SO_3_^−^	H	–	Na^+^
**21**	**V**	a/a’	H	CH_3_	H	–	ClO_4_^−^
**22**	**V**	a/a’	Ph-O-	(CH_2_)_4_SO_3_^−^	SO_3_^−^	–	3Na^+^
**23**	**IV**	a/a’	Ph-O-	C_4_H_9_	H	–	PF_6_^−^
**24**	**V**	b/b’	Barb1	C_2_H_5_	H	–	–
**25**	**IV**	a/a’	Ph-O-	(CH_2_)_4_SO_3_^−^	H	–	Na^+^
**26**	**IV**	a/a’	Barb2	CH_3_	H	–	–
**27**	**IV**	a/a’	Barb2	C_4_H_9_	H	–	–
**28**	**V**	a/a’	Ph-	CH_3_	H	–	Cl^−^
**29**	**IV**	a/a’	Barb2	(CH_2_)_4_SO_3_^−^	H	–	2 Na^+^
**30**	**V**	a/a’	Ph-O-	CH_3_	H	–	Cl^−^
**31**	**IV**	a/a’	Ph-	C_4_H_9_	SO_3_^−^	–	Na^+^
**32**	**IV**	a/a’	Ph-	(CH_2_)_4_SO_3_^−^	SO_3_^−^	–	3 Na^+^
**33**	**V**	a/a’	Barb1	iC_5_H_11_	Cl	–	–
**34**	**IV**	b/b’	Barb2	CH_3_	H	–	–
**35**	**IV**	a/a’	(Ph)_2_N-	C_4_H_9_	H	–	PF_6_^−^
**36**	**IV**	a/a’	(Ph)_2_N-	C_4_H_9_	H	–	BF_4_^-^
**37**	**IV**	a/a’	(Ph)_2_N-	C_4_H_9_	H	–	[Al(O-*t*-C_4_F_9_)_4_]^−^
**38**	**V**	a/a’	Ph(Ph-CH_2_)N-	C_4_H_9_	H	–	PF_6_^−^
**39**	**IV**	a/a’	(Ph)_2_N-	(CH_2_)_4_SO_3_^−^	H	–	Na^+^
**40**	**IV**	a/a’	(Ph)_2_N-	(CH_2_)_4_SO_3_^−^	SO_3_^−^	–	3Na^+^
**41**	**IV**	a/a’	(Ph)_2_N-	(CH_2_)_4_SO_3_^−^	Cl	–	HN(C_2_H_5_)_3_^+^
**42**	**IV**	a/a’	Barb2	C_4_H_9_	SO_3_^−^	–	2 Na^+^
**43**	**V**	a/a’	Barb2	C_4_H_9_	H	–	–
**44**	**IV**	a/a’	Cl	C_4_H_9_	H	–	BF_4_^−^
**45**	**V**	a/a’	Cl	(CH_2_)_4_SO_3_^−^	SO_3_^−^	–	3 Na^+^
**46**	**IV**	a/a’	Cl	CH_3_	H	–	MePhSO_3_^−^
**47**	**V**	a/a’	Barb2	C_2_H_5_	H	–	–
**48**	**V**	b/b’	Barb2	C_2_H_5_	H	–	–
**49**	**V**	a/a’	Barb2	(CH_2_)_4_SO_3_^−^	H	–	2 Na^+^
**50**	**IV**	a/a’	Barb2	(CH_2_)_4_SO_3_^−^	SO_3_^−^	–	4 Na^+^
**51**	**V**	b/b’	Barb4	C_2_H_5_	H	–	–
**52**	**IV**	a/a’	Cl	C_4_H_9_	SO_3_^−^	–	Na^+^
**53**	**IV**	a/a’	Cl	(CH_2_)_4_SO_3_^−^	H	–	Na^+^
**54**	**V**	a/a’	Cl	(CH_2_)_4_SO_3_^−^	H	–	Na^+^
**55**	**V**	a/a’	Cl	C_4_H_9_	H	–	PF_6_^−^
**56**	**IV**	a/a’	Cl	(CH_2_)_4_SO_3_^−^	SO_3_^−^	–	3 Na^+^
**57**	**V**	a/a’	Cl	C_4_H_9_	Cl	–	PF_6_^−^
**58**	**V**	a/a’	Cl	C_4_H_9_	SO_3_^−^	–	Na^+^
**59**	**III**	b/b’	H	(CH_2_)_4_SO_3_^−^	H	–	Na^+^
**60**	**V**	a/a’	Tria	C_4_H_9_	H	–	BF_4_^−^
**61**	**IV**	a/a’	Ph-S-	(CH_2_)_4_SO_3_^−^	H	–	Na^+^
**62**	**V**	a/a’	Barb3	CH_3_	H	–	–
**63**	**V**	a/a’	Cl	CH_3_	H	–	Cl^−^
**64**	**V**	a/a’	Ph-S-	CH_3_	H	–	Cl^−^
**65**	**V**	a/a’	Ph-S-	C_4_H_9_	H	–	ClO_4_^−^
**66**	**V**	a/a’	Ph-S-	(CH_2_)_4_SO_3_^−^	H	–	Na^+^
**67**	**V**	a/a’	Ph-S-	(CH_2_)_4_SO_3_^−^	SO_3_^−^	–	3 Na^+^
**68**	**IV**	a/a’	Ph-S-	(CH_2_)_4_SO_3_^−^	SO_3_^−^	–	3 Na^+^
**69**	**V**	a/a’	C_6_H_13_	(CH_2_)_4_SO_3_^−^	SO_3_^−^	–	3Na^+^
**70**	**V**	a/a’	Barb2	(CH_2_)_4_SO_3_^−^	SO_3_^−^	–	4 Na^+^
**71**	**V**	a/a’	Barb5	(CH_2_)_4_SO_3_^−^	SO_3_^−^	–	4 Na^+^
**72**	**V**	a/a’	(Ph)HN-	C_4_H_9_	H	–	PF_6_^−^
**73**	**V**	a/a’	(Ph)HN-	(CH_2_)_4_SO_3_^−^	H	–	Na^+^
**74**	**V**	a/a’	IM^+^	C_4_H_9_	H	–	2 PF_6_^−^
**75**	**V**	a/a’	CH_3_	(CH_2_)_4_SO_3_^−^	SO_3_^−^	–	3Na^+^
**76**	**V**	a/a’	C_6_H_13_	CH_3_	H	–	ClO_4_^−^
**77**	**V**	a/a’	CH_3_	CH_3_	H	–	Cl^−^
**78**	**V**	a/a’	PMT	CH_3_	H	–	Cl^−^
**79**	**V**	a/a’	PMT	iC_5_H_11_	H	–	DoPhSO_3_^−^
**80**	**V**	a/a’	PMT	C_2_H_5_	Cl	–	NTf_2_^−^
**81**	**V**	a/a’	PMT	C_2_H_5_	Cl	–	DoPhSO_3_^−^
**82**	**V**	a/a’	PMT	C_2_H_5_	Cl	–	ViPhSO_3_^−^
**83**	**V**	b/b’	PMT	C_2_H_5_	H	–	NTf_2_^−^
**84**	**V**	a/a’	IM	C_4_H_9_	H	–	PF_6_^−^
**85**	**V**	a/a’	(Ph)HN-	C_4_H_9_	Cl	–	PF_6_^−^
**86**	**V**	a/a’	(Ph)HN-	(CH_2_)_4_SO_3_^−^	SO_3_^−^	–	3 Na^+^
**87**	**V**	a/a’	(Ph)HN-	C_4_H_9_	SO_3_^−^	–	Na^+^

^a^See Scheme 1. ^b^See Scheme 2. ^c^Structure elements of Table 1:
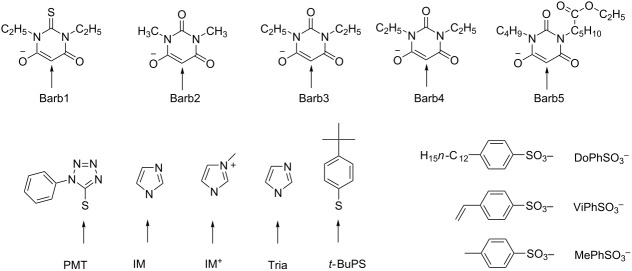

**Table 2:** Summary of photophysical (absorption maximum λ_max_^abs^, fluorescence maximum λ_max_^flu^, extinction coefficient ε, fluorescence quantum yield Φ_f_) and electrochemical data (oxidation potential *E*_ox_, and reduction potential *E*_red_). Data of absorbers^a^ relate to the structural patterns **I**–**V**. Supporting Information File 1 gives information of the electrochemical measurements (data were taken in CH_3_CN), and the determination of photophysical data. The latter complements previously published data [5–6] helping to receive a more completed pattern about these NIR absorbers.

#	λ_max_^abs^ (nm)	λ_max_^flu^ (nm)	Φ_f_ (%)	ε (10^5^ × M^−1^ cm^−1^)	solvent	*E*_ox_ (V)	*E*_red_ (V)	Reference

**1**	758	777	0.5	1.26	MeOH			^b^
**2**	858	877	0.3	2.09	MeOH	0.73	−0.27	^b^
**3**	916				MeOH			^b^
**4**	946	986	0.1	2.07	MeOH	0.69	−1.01	[5]
**5**	973			2.08	MeOH			^b^
**6**	995	^c^	0.001	1.76	MeOH	0.61	−0.25	[5]
**7**	1024				MeOH			^b^
**8**	1024				MeOH			^b^
**9**	997				MeOH			^b^
**10**	997		0.001	2.23	MeOH	0.66	−0.34	[5]
**11**	1014				MeOH			^b^
**12**	746	774	26	2.80	MeOH			^b^
**13**	752 748	781 774	25 15	2.40	MeOH H_2_O			^b^
**14**	766	784	20	3.04	MeOH			^b^
**15**	768	786	19	2.43	MeOH			^b^
**16**	740	766	19	2.22	MeOH			^b^
**17**	657	783	19	1.29	MeOH			^b^
**18**	760	778	19	3.28	MeOH			^b^
**19**	767 759	785 778	19 9	3.02 2.26	MeOH H_2_O			^b^
**20**	761	779	18	2.41	MeOH			^b^
**21**	744	765	18	2.08	MeOH			^b^
**22**	772 769	790 789	17 7	1.61 1.38	MeOH H_2_O			^b^
**23**	790	812	16	3.13	MeOH			^b^
**24**	792	815	16	2.88	MeOH	0.49	−1.11	[5]
**25**	790	812	15	2.63	MeOH			^b^
**26**	777	800	15	3.58	MeOH	0.40	−0.80	[5]
**27**	784	806	15	3.00	MeOH			^b^
**28**	755	772	15	2.79	MeOH			^b^
**29**	784	806	14	2.57	MeOH			^b^
**30**	763	781	14	2.34	MeOH			^b^
**31**	796	817	14	2.98	MeOH			^b^
**32**	796 790	818 813	13 6	2.84 2.30	MeOH H_2_O			^b^
**33**	763	787	13	3.15	MeOH	0.47	−0.87	[5]
**34**	811	834	13	3.17	MeOH	0.42	−0.87	[5]
**35**	794	818	13	2.60	MeOH			^b^
**36**	792		^d^	2.41	MeOH	0.57	−0.60	[5]
**37**	^d^	^b^	^d^			^d^	^d^	[6]
**38**	795	821	13	2.00	MeOH			^b^
**39**	796	819	11	1.57	MeOH	^d^	^d^	^b^
**40**	796 786	824 818	11 2	2.14 1.72	MeOH H_2_O	^d^	^d^	^b^
**41**	796		11	1.63	MeOH	0.60	−0.57	[5]
**42**	788	810	11	2.44	MeOH	^d^	^d^	^b^
**43**	759	781	11	2.92	MeOH	^d^	^d^	^b^
**44**	804	823	11	2.39	MeOH			^b^
**45**	789 782	807 800	11 5	2.49 2.15	MeOH H_2_O			^b^
**46**	773	797	10	1.97	MeOH	0.68	−0.50	[5]
**47**	756	778	10	2.62	MeOH	0.41	−0.86	[5]
**48**	791	811	10	2.76	MeOH	0.48	−0.97	[5]
**49**	759 760	781 780	10 6	2.17 1.70	MeOH H_2_O	^d^	^d^	^b^
**50**	790 790	811 811	10 6	1.96 1.50	MeOH H_2_O	^d^	^d^	^b^
**51**	791	810	10	2.80	MeOH	^d^	^d^	^b^
**52**	812	831	10	2.66	MeOH	^d^	^d^	^b^
**53**	806	825	10	2.66	MeOH	^d^	^d^	^b^
**54**	782	801	10	2.63	MeOH	^d^	^d^	^b^
**55**	781	800	10	3.07	MeOH	0.68	−0.50	[5]
**56**	813 804	832 824	9 4	2.41 2.27	MeOH H_2_O	^d^	^d^	^b^
**57**	790	807	9	2.89	MeOH	^d^	^d^	^b^
**58**	788	807	9	2.57	MeOH	^d^	^d^	^b^
**59**	784	819	9	2.33	MeOH			^b^
**60**	791	809	9	2.81	MeOH			^b^
**61**	820	842	8	2.78	MeOH	^d^	^d^	^b^
**62**	753	774	8	2.72	MeOH	^d^	^d^	^b^
**63**	775	792	7	2.37	MeOH	^d^	^d^	^b^
**64**	787	810	7	2.41	MeOH	0.75	−0.49	[5]
**65**	793	815	7	2.77	MeOH			^b^
**66**	795 788	819 814	7 3	2.33	MeOH H_2_O			^b^
**67**	801 794	824 818	6 3	2.46 2.04	MeOH H_2_O			^b^
**68**	827 819	850 842	6 3	2.24 2.29	MeOH H_2_O			^b^
**69**	781	799	6	2.51	MeOH			^b^
**70**	765 766	786 785	6 6	2.33 2.18	MeOH H_2_O			^b^
**71**	765	785	6	2.55	MeOH			^b^
**72**	745	791	6	1.66	MeOH			^b^
**73**	745	791	5	0.12	MeOH			^b^
**74**	798	816	5	2.13	MeOH			^b^
**75**	775 767	797 789	4 3	2.42 1.98	MeOH H_2_O			^b^
**76**	768	787	4	2.70	MeOH			^b^
**77**	763	785	3	2.60	MeOH			^b^
**78**	799	817	3	2.26	MeOH			^b^
**79**	813	^d^	^d^	1.62	MeOH	^d^	^d^	^b^
**80**	810	^d^	^d^	^d^	MeOH	^d^	^d^	^b^
**81**	807	^d^	^d^	2.40	MeOH	0.52	−0.52	[5]
**82**	809	^d^	^d^	2.13	MeOH	0.64	−0.52	[5]
**83**	844		^d^	1.35		0.56	−0.57	[5]
**84**	785	803	2	2.93	MeOH	0.67	−0.51	[5]
**85**	744	800	2	1.43	MeOH			^b^
**86**	739 731	795 788	1 0.1	1.01 0.90	MeOH H_2_O			^b^
**87**	736	795	0.8	1.10	MeOH			^b^

^a^FEW Chemicals GmbH provided those materials as samples, which were not previously investigated. ^b^Data were taken in this work to complement previously published data [5–6]. See Supporting Information File 1 for experimental details. ^c^Not possible to determine. ^d^Not determined due to similarity of above explored cyanine pattern, similar quantity was expected.

Absorbers comprising indolium and benzo[*e*]indolium derivatives resulted in materials exhibiting an observable fluorescence. The fluorescence quantum yield (Φ_f_) obtained approached for some absorbers around 25%. This related to materials comprising the general pattern **III**. The Φ_f_-data sometimes dropped by changing the substituent at the indolium nitrogen resulting in a decrease of the molar extinction coefficient (ε) as well. Aggregation/dimerization can cause such phenomena, which typically leads to a decrease of absorption at the considered absorption maximum and a pronounced non-radiative deactivation resulting in a decrease of Φ_f_. Moreover, structures based on **IV** and **V** additionally provided absorbers with fluorescence quantum yields of up to 20% yield. Cyanines comprising at the *meso*-position either a phenyl or phenoxy group resulted in a Φ_f_ of about 15–20% (derivatives comprising PhO-: **14**, **15**, **22**, and **30**; derivatives comprising Ph-: **18** – **20**, **23**, **25**, **28**, **31**, and **32**). Absorbers with a barbiturate group at the same position resulted in fluorescence quantum yields between 10–16% (compare **24**, **26**, **27**, **29**, **33**, **34**, **42**, **43**, **47**–**51**). Diphenylamino substitution at the *meso*-position showed a slight decrease of Φ_f_ (compare **35**). Replacement with thiol moieties such as phenylmercapto and phenylmercapto-tetrazole substituents resulted in Φ_f_ of 7% (compare **64**–**68**) and 3% (compare **78**), respectively. Thus, one can roughly draw the following order for the decrease fluorescence quantum yields located at the *meso*-position:

PhO-; Ph- ≥ barbiturate ≥ Ph_2_N- > PhS- > phenylmercapto tetrazole.

Thus, it would be difficult to draw conclusions based on these substituents with respect to their electronic properties. While Ph-O, Ph_2_N-, and Ph-S- exhibit electron donating properties, barbiturate and phenyl substituents can be seen more or less as electron withdrawing moieties. Quantum chemical calculations showed only small contributions of the substituent placed at the *meso*-position to the electron density in the HOMO/LUMO pattern [81]. Presumably, coupling of lower occupied molecular orbitals with the HOMO on the one hand side and higher unoccupied MOs with the LUMO on the other side may give an answer regarding the electronic interaction of this substituent with the methine chain.

In general, these absorbers combine interesting features; that is emission on one hand side and the release of heat on the other side. The latter uptakes the major part; that is more than 80–95% depending on substitution. Thus, such absorbers can also work in systems where typically thermal processes initiated by furnace techniques play the major role. These absorbers generate heat on demand just by switching on and off the light source. Thus, they can be seen more or less as aforementioned molecular ovens. Recently development of light sources in the NIR based on LEDs brought new hope in this field [65]. Furthermore, the fact that still some fluorescence occurs makes them interesting as fluorescent probes for imaging applications. NIR radiation possesses a scattering coefficient being a third with respect to those measured at 400 nm [82]. Particularly, biological materials have received increased interest since they benefit from deeper penetration of radiation into the sample.

Absorbers with an absorption maximum >900 nm (compare **2**–**11**) exhibit fluorescence quantum yields of <<1% showing that more than 99% of the absorbed light energy was converted to heat. From this point of view, they appear more or less as efficient molecular ovens, which was practically approved by melting of powder coatings applying lasers with line-shaped focus [16]. Thus, these absorbers possess large potential to replace thermal processes based on furnaces by introduction of such molecular ovens in combination with modern light sources [64–65,67]. The development of modern LED systems with emission in the NIR will enforce such developments [64].

Compatibility with the surroundings is the biggest challenge in practical applications of these systems. Thus, counter ions such as *n*-C_12_H_25_-Ph-SO_3_^−^ (**8**, **10**, **79**, **81**), NTf_2_^−^ (**4**) or [Al(OC_4_F_9_)_4_]^−^ (**37**) depict alternatives resulting in a solubility of 10–30 g/L in reactive monomers such as multifunctional acrylates [5], printing inks or powder coatings. Alternatively, the introduction of alkyl groups with branched moieties (**79**) interestingly improved solubility with the surrounding matrix as well [5]. As aforementioned noticed, this area appears more or less as semi-empirically disclosed and would require even more theoretical work based on molecular modeling to understand relationships/interactions between absorbers and a surrounding matrix in the near future.

The introduction of functional groups promoting the solubility in water results in absorbers exhibiting an appropriate water solubility, see Table 1 for structures. This can be either -N^+^RR’R’’ or a SO_3_^−^ group. The fluorescence quantum yields obtained in water exhibit smaller values compared to those derivatives with no SO_3_^−^ groups taken in MeOH. Nevertheless, the emission released should appear large enough for imaging application of biological samples.

### Photoinduced electron transfer and internal barriers

Sensitized generation of reactive intermediates such as radicals and conjugate acid [5] also followed in the case of NIR sensitive materials a photoinduced electron transfer (PET). However, it did not work as smooth as disclosed for UV systems [83] because there was often no reaction between a cationic sensitizer and an electron acceptor; i.e. an iodonium salt [5]. However, the use of stronger emitting LEDs resulted sometimes in successful PET [67]. Particularly, light sources with low emission intensity arose these issues. This supports the idea that the system possesses an internal activation barrier resulting in a system having a certain energy threshold. Equation 8 shows how temperature affects the rate constant for electron transfer *k*_et_ The free activation enthalpy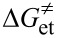 [67,72] controls the internal activation barrier, Equation 8 [65]. The free enthalpy of electron transfer (Δ*G*_et_) and the reorganization energy λ depict additional parameters affecting *k*_et_. Knowledge about redox potentials and excitation energy provides information about Δ*G*_et_ needed for Equation 8 [72]. The dielectric constant ε and refractive index *n* of the surrounding matrix contribute to the outer sphere coordinates (λ_o_) while geometric changes of the starting materials responsible tune inner-sphere coordinates λ_i_ (λ = λ_o_ + λ_i_) [72].

[8] $$k_{\text{e}}{}_{\text{t}}=\nu_{N}\cdot \kappa \times exp\left(-\frac{\Delta G_{\text{et}}^{\neq }}{RT}\right)=\nu_{N}\cdot \kappa \times exp\left(-\frac{{\left(\Delta G_{\text{et }}^{\neq }+\lambda \right)}^{2}/4\lambda }{RT}\right)$$

**Scheme 5:**
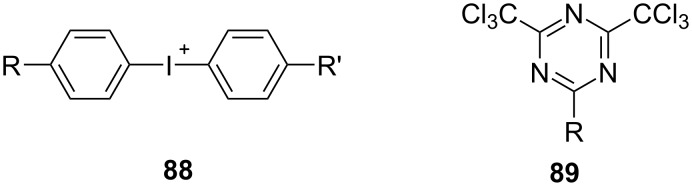
Chemical structure of the electron acceptors that were from iodonium cations **88** and triazines **89**.

Many NIR sensitive systems possess an internal activation barrier although Δ*G*_et_ < 0 using either the positively charged iodonium compound **88** (*E*_red_ = −0.64 V [5,84]) or the triazine **89** (*E*_red_ = −0.77 V [5]) carrying no charge as acceptor (Scheme 5). Thus, the positive charge of the iodonium cation is likely not the reason because **89** exhibits no charge. Both possess similar reduction potentials but the efficiency to initiate radical polymerization was higher in the case of **88** [5]. Experimentally, there is often no reaction under ambient light conditions. However, strong sources such as lasers with line-shaped focus or high-power NIR-LEDs helped to initiate photochemical reactions [64–65,67] . Recent studies confirmed these findings [85–88].

**Figure 2:**
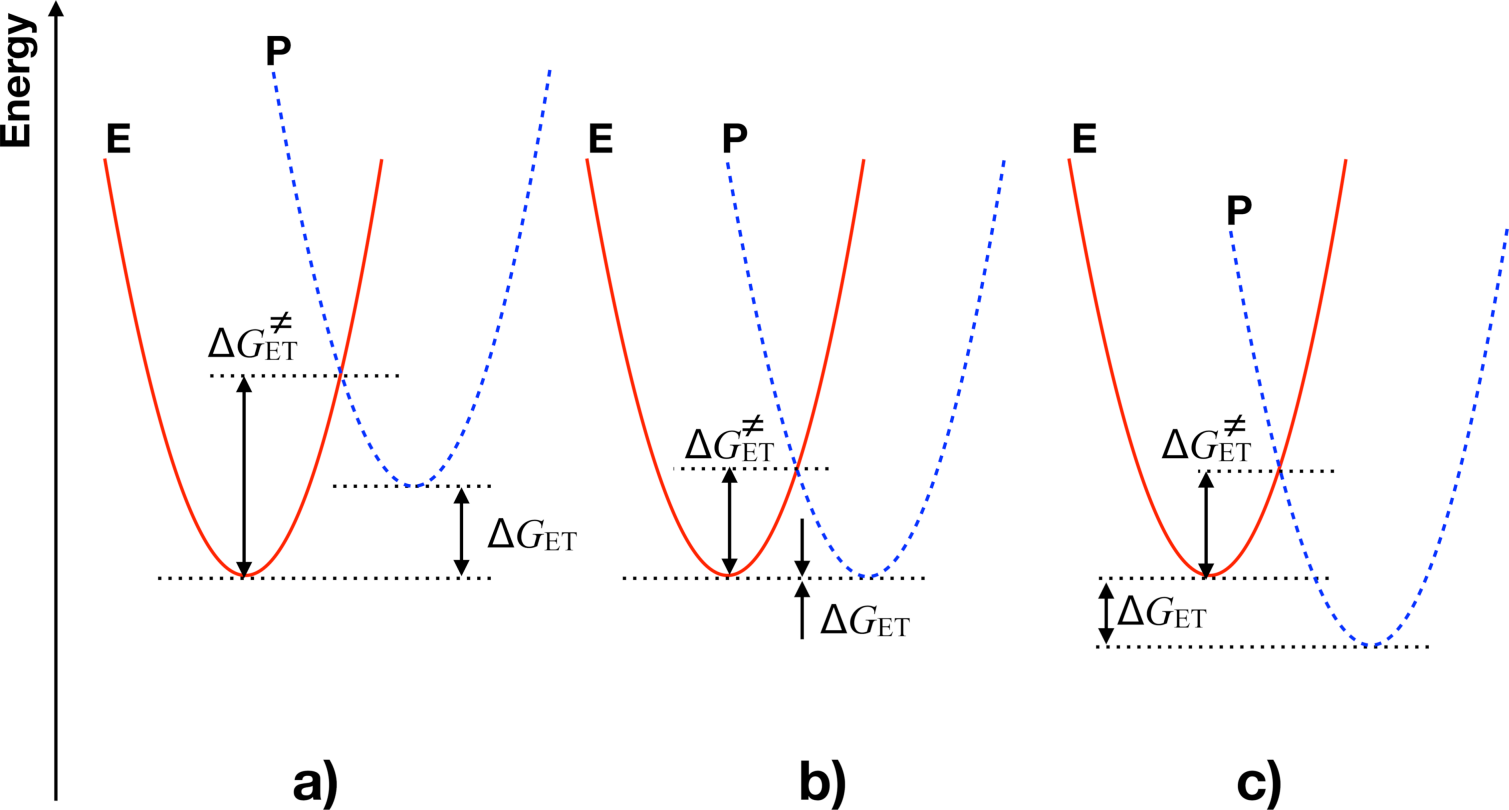
Photoinduced electron transfer under different scenarios in which each example exhibits an intrinsic activation barrier resulting in threshold systems a) endothermal conditions, b) thermoneutral conditions, c)exothermal conditions.

Crossing of the potential energy curves of the starting materials comprising **Sens** and **88** with those of the products (**Sens**^+•^ and **88**^−•^) results in the scenario shown in Figure 2 [65,67]. It requires under some circumstances to introduce additional heat also if Δ*G*_et_ < 0 to cross the intrinsic barrier. NIR-sensitive systems comprising **12**–**87** and **13** or **14** result in Δ*G*_et_ being +/−0.5 eV using **88** as acceptor [5]. The reorganization energy λ can approach values of 1–1.5 eV [65,70]. This helps to overcome the internal activation barrier. Non-radiative deactivation of the NIR absorber provides enough thermal energy to overcome the activation barrier. It can easily achieve temperatures >100 °C and may be certainly seen as a new concept also to drive thermally controlled reactions [56]. Again, the intrinsic activation barrier exhibits in all three examples a similar value while a) relates to endothermal conditions, b) corresponds to thermoneutral conditions and c) displays the scenario for exothermal conditions. The inverse case with extremely large Δ*G*_et_ is not considered in this example. It also relates to large intrinsic 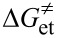.

Only some of the absorbers showed chemical reactivity in the case of a low intensity (<100 mW/cm^2^) NIR-LED resulting in radicals and conjugate acid [5]. Most of them comprised a barbiturate group. **26**, **34**, **47**, and **48** depict some representative examples. On the other hand, positively charged sensitizers showed no reactivity with such a low intensity LED [5]. Switching the LED source to a device providing significant higher exposure intensity resulted in a remarkable reactivity of even positively charged sensitizers [65]. This helped the system to travel over the internal activation barrier and facilitated access to photopolymerization reactions. Moreover, it also improved handling of such NIR sensitive materials under ambient room light conditions where the formulations appeared as stable.

**Scheme 6:**
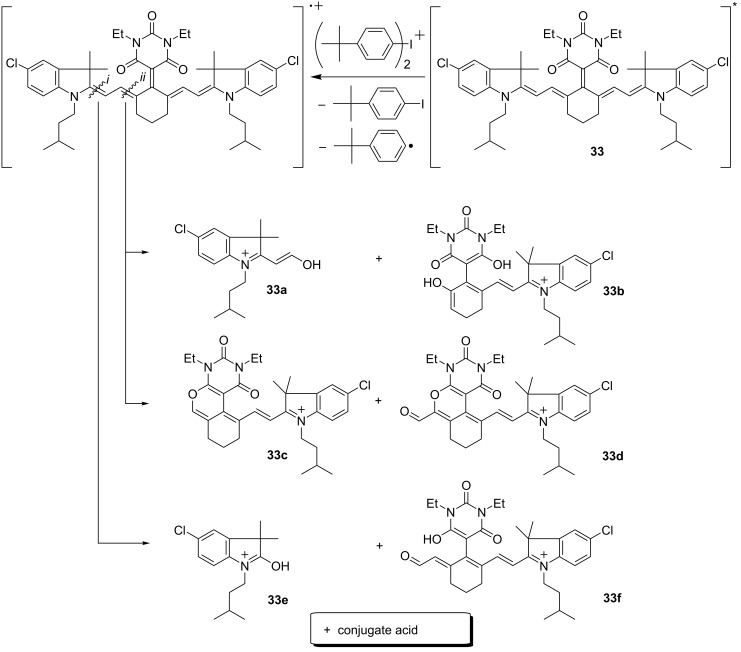
Photoexcited absorber **33** results in reaction with an iodonium cation in the respective cation radical. Cleavage in the methine chain leads to the reaction products **33a**–**f**.

Future developments should also focus on systems resulting in less yellowing upon exposure in the presence of an acceptor. As aforementioned discussed, the brown color formed may be seen as an issue. Particularly **48** showed remarkable polymerization efficiency in combination with **88** [5,63]. Photoinduced electron transfer typically followed an exothermal reaction route resulting in the formation of the cation radical of the sensitizer and the iodyl radical (Ar_2_-I^•^) formed by reduction of the diaryl iodonium salt, Scheme 6. This occurred from the singlet state while no indication has been available regarding the involvement of triplet states [89]. Sometimes singlet oxygen was believed to play a major role to explain the bleaching of cyanines [90–91]. This report does not consist with our findings where no formation of singlet oxygen was confirmed [89]. Other pathways request attention which could be formation of the O_2_^-•^ radical to propose an alternative [90]. Such intermediates exhibit high reactivity resulting in similar photoproducts as disclosed for photoinduced electron transfer vide infra [63]. The cation radical formed cleaved at the methine chain resulting in the reaction products **33a**–**f**. They appear as yellow/brownish materials and cause the partially unpleasant color appearance of thick clear coats.

Furthermore, the reaction products comprise nucleophilic nitrogen that inhibits cationic polymerization of epoxides [5]. Though conjugate acids are formed according to the routes shown in Scheme 6, cationic photopolymerization only succeeded with aziridines [5]. The different reaction mechanism occurring with such monomers explains these different observations [92]. Aziridines tolerate nucleophiles because chain growth requests the ammonium ion [93–96]. On the other hand, chain propagation includes the carbocation in the case of oxiranes and oxetanes [92].

**Scheme 7:**
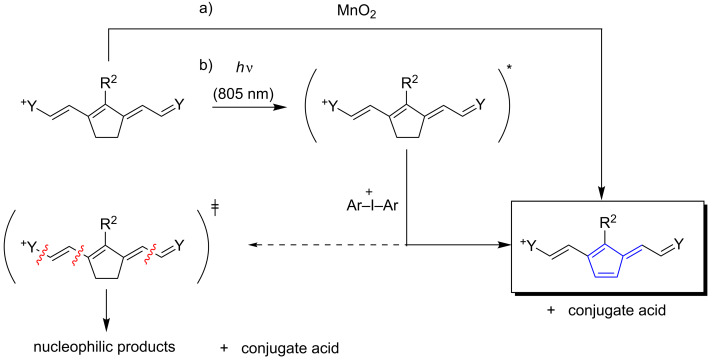
Reaction scheme of absorbers comprising in the molecules center a five ring bridged moiety. This leads to two competitive pathways (from [65]. Schmitz, C.; Pang, Y.; Gülz, A.; Gläser, M.; Horst, J.; Jäger, M.; Strehmel, B., New High-Power LEDs Open Photochemistry for Near-Infrared-Sensitized Radical and Cationic Photopolymerization. *Angew. Chem., Int. Ed. ***2019,** *58* (13), 4400-4404. Copyright Wiley-VCH Verlag GmbH & Co. KGaA. Reproduced with permission).

More of interest can be seen absorbers, which do not follow such reaction routes. Scheme 7 depicts a general structure following not mainly the path shown in Scheme 7. They derive from the general structural pattern **IV** comprising a five-membered moiety in the center of the conjugated system. Compounds **35**–**37** represent some absorbers fitting in this scheme [65]. Particularly, the five-membered moiety opens new deactivation channels, which is the formation of an additional double bond [95–96]. This leads to a change of the conjugated system from a cyanine motif to a fulvene type. The latter shows, although if it contains one additional double bond and thus two additional π-electrons more, a hypsochromic shifted absorption of about 100 nm. Practically, such systems appear blue after exposure, which brings them to color on demand applications [97–98]. Furthermore, cleavage of the methine chain resulting in nucleophilic products seems to occur with minor importance. This explains why no brownish photoproducts were formed and cationic photopolymerization even of epoxides succeeded with good conversions vide infra. Additional absorbers fitting in this scheme comprise at the *meso*-position alternative substituents such as Phenyl or Chlorine (see Table 1 for comparison). Surprisingly, this reaction has not received remarkable attention in the academic community although industrial applications use this reaction to pattern imaged areas [97–98]. The structure was confirmed by direct synthesis following a procedure using MnO_2_[63].

### Polymer synthesis

#### NIR sensitized radical photopolymerization

Photosensitized formation of reactive intermediates such as initiating radicals follows the Equations 1–7 vide supra. The system comprises as sensitizer an absorber derived from structure **IV** or **V**, and an electron acceptor such as **88** or **89**. The main challenge can be seen to drive the system somehow that Equation 5 relating to electron back transfer occurs with minor importance. As vide infra mentioned, involvement of triplet states in such sensitized systems, which could decrease the rate for this reaction, does not play a major role. Thus, no change of spin multiplicity may cause a high rate of Equation 5 as long as the systems remains in the singlet state. There must be found an opportunity to make the system irreversible so that the electron back transfer in Equation 5 receives minor importance. Thus, cleavage of the main chain [63] (Scheme 6) or formation of distinct conjugated patterns [65] (Scheme 7) depict alternative reaction routes. In this case, the formation of initiating radicals follows an oxidative mechanism with respect to the sensitizer resulting in irreversibility of the system.

**Scheme 8:**
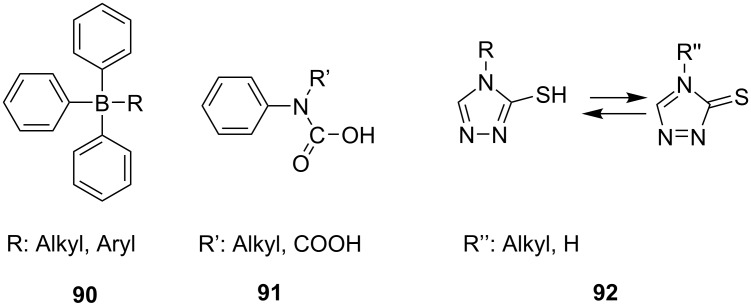
Structure of donor compounds used in a three component system.

Sometimes, it was practiced adding a third component into the system exhibiting electron donating properties to keep the efficiency of Equation 5 on a low level. Thus, the donor transfers an electron into the SOMO of the **Sens**^+•^, Equation 6, resulting in back formation of the **Sens**. The oxidized donor unimolecular cleaves in a consecutive reaction resulting in formation of additional initiating radicals. Typical donors applied in such systems are borates **90** [99–114], phenyl imino acetic acid **91** [115] or mercapto triazoles **92** [116]. The latter often exists in its tautomeric thione form. Scheme 8 shows the respective structures.

References [11–14,63–65] provide more details regarding the mechanistic details. Such systems have been successfully applied in CtP systems for more than 20 years [11–12]. Some pioneering works also disclose their use for chemical solidification of varnishes [5]. Remarkable has been still the function in powder coating, where they properly helped to melt and cure the applied powder monomers nearly just in one step [16]. Typical monomers used in CtP, liquid coatings and powder coatings derive from urethane (meth)acrylates, epoxy (meth)acrylates, polyester(meth)acrylates or polyether (meth)acrylates. Literature provides some reviews and original articles discussing mechanistic details [5–6,11–14,54,63–64,67,85].

#### NIR sensitized cationic photopolymerization

Equations 1–7 responsibly affect the efficiency of cationic photopolymerization. This was first demonstrated in 2016 [5] taking a combination of the sensitizer **48** and the iodonium cation **88** (used as PF_6_^−^-salt) to photopolymerize aziridines. The photoproducts formed according to Scheme 6 do not significantly inhibit chain growth of such monomers while no polymerization occurred with epoxides. Again, the strong nucleophilic properties of the photoproducts **33a**–**f** did not permit successful run of cationic polymerization neither of oxiranes nor oxetanes. This became possible with the sensitizers **35**–**37** differing only with respect to their anion; that is [BF_4_]^−^, [PF_6_]^−^ and [Al(O-*t*-C_4_F_9_)]^−^ [6] after the first successful report of NIR-sensitized cationic photopolymerization of epoxides [65]. Though [Al(O-*t*-C_4_F_9_)_4_]^−^ possesses the lowest nucleophilicity in this series, its high molecular weight requires a higher loading to obtain similar OD compared with sensitizers carrying anions with lower molecular weight. There exists still the issue regarding HF formation in the case of [PF_6_]^–^-salts while no issues have been reported for the aluminate [6]. The oxidized intermediate **PA**^+•^ (Equation 7) forms the conjugate acid by decomposition of this instable intermediate resulting in nucleophilic photoproducts inhibiting ring opening polymerization mechanism where carbocations are involved.

**Figure 3:**
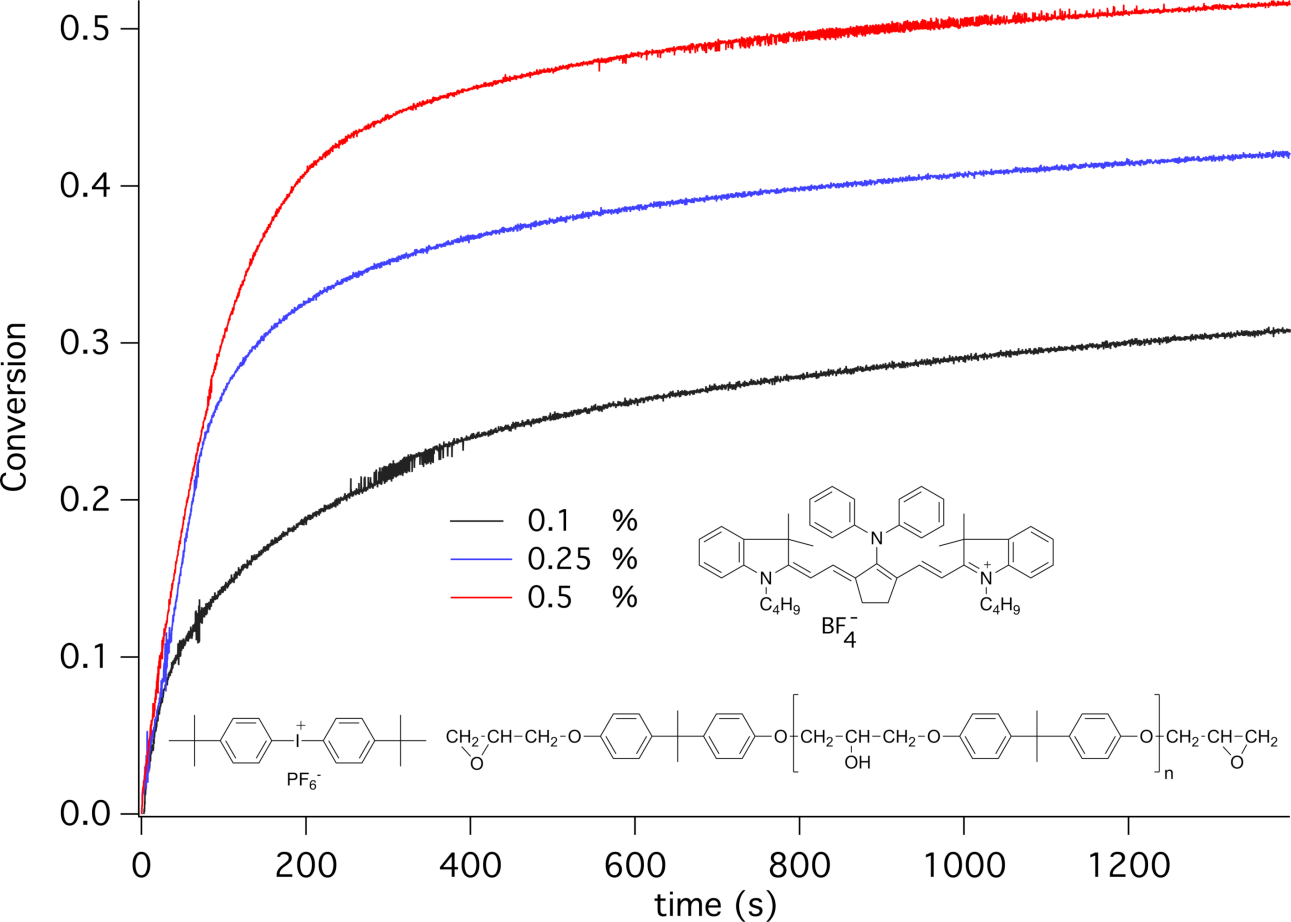
Cationic photopolymerization of an epoxide (Epikote 828) initiated by excitation of the absorber **36** and bis(4-*tert*-butyl phenyl)iodonium hexafluorophosphate (2 wt %) applying different NIR sensitizer concentrations with an NIR-LED source emitting at 805 nm with an intensity of 1.2 W cm^–2^. (From [65]. Schmitz, C.; Pang, Y.; Gülz, A.; Gläser, M.; Horst, J.; Jäger, M.; Strehmel, B., New High-Power LEDs Open Photochemistry for Near-Infrared-Sensitized Radical and Cationic Photopolymerization. *Angew. Chem., Int. Ed. ***2019,** *58* (13), 4400–4404. Copyright Wiley-VCH Verlag GmbH & Co. KGaA. Reproduced with permission).

Another strategy followed Scheme 7 in which formation of a fulvene like pattern prevented formation of nucleophilic photoproducts. This five ring pattern in the molecules center surprisingly prevents cleavage of the methine chain resulting in the oxidation mechanism in a fulvene pattern. The nitrogens in the cyanine chain still exhibit low nucleophilicity since electron density distributes over the entire methine chain. Thus, protonation of the nitrogen in the cyanine moiety occurs with less efficiency compared to amines [117]. This strategy helped to enable cationic photopolymerization with cyanine as sensitizers combined with **88** as PF_6_^−^-salt. Exposure with a high power NIR LED emitting at 805 nm initiated cationic photopolymerization of the epoxide Epikote 357, Figure 3. Decomposition of the oxidized sensitizer/oxidized photoactive compound **PA**^+•^ (Equation 7) provided the conjugate acid needed for initiation. Thus, the higher the concentration of **PA**, the higher the conversion. Figure 3 nicely documents this scenario.

In 2019, the first report about NIR-sensitized cationic photopolymerization appeared in combination with a high intensity NIR LED [65]. Such a combination facilitated the NIR photoinitiating system to move over the internal reaction barrier as explained in Figure 2. Until this time, NIR LEDs exhibiting low intensity served as exposure source where only **36** showed photochemical activity in combination with an iodonium cation. The fact that such systems comprising a cationic absorber as sensitizer can also react was not recognized until this time since strong emitting devices were not available. This technology based on NIR absorbers possess big potential in the near future since the development of more devices with emission centering between 800–1000 nm has been in progress.

#### Tailor-made synthesis by photo-ATRP with NIR sensitizers

NIR absorbers/sensitizers have also reached the field of polymer synthesis; that is controlled/living radical polymerization (CLRP). This facilitates well-defined polymeric materials with pre-design architectures, predetermined molar masses and narrow molecular weight distributions. These techniques allow linear increase of molar mass by time and monomer conversion and low dispersity (principally between 1.2 to 1.5). The most common methods of controlled radical polymerization are namely the nitroxide-mediated radical polymerization (NMP) processes, reversible addition-fragmentation chain transfer (RAFT) and atom transfer radical polymerization (ATRP) [118]. While there has been no report available with NIR-sensitized NMP, there exist a few reports for RAFT polymerization with NIR light [119–121]. Recently, ATRP with Cu(II)-catalyst scaling in ppm amount in combination with a NIR sensitizer facilitated tailor-made synthesis of block copolymers [81]. This helped to reduce the heavy metal concentration in the polymerization system at a low level and complements therefore several methods developed to operate at low catalyst concentration by continuous regeneration of Cu(I) species using Cu(II) as starting material. These methods include initiators for continuous activator regeneration (ICAR) ATRP, activators regenerated by electron transfer (ARGET) ATRP, supplemental activators and reducing agent (SARA) ATRP, and electrochemically mediated ATRP (eATRP) [122–128]. In this content, various reducing agents, and electrochemical redox processes, and copper comprising nanoparticles have been accomplished to conduct ATRP by generating the required Cu(I) by the reduction of Cu(II) complexes. A well written review provides available references disclosing controlled polymer synthesis via photoinduced electron transfer reactions [129].

**Scheme 9:**
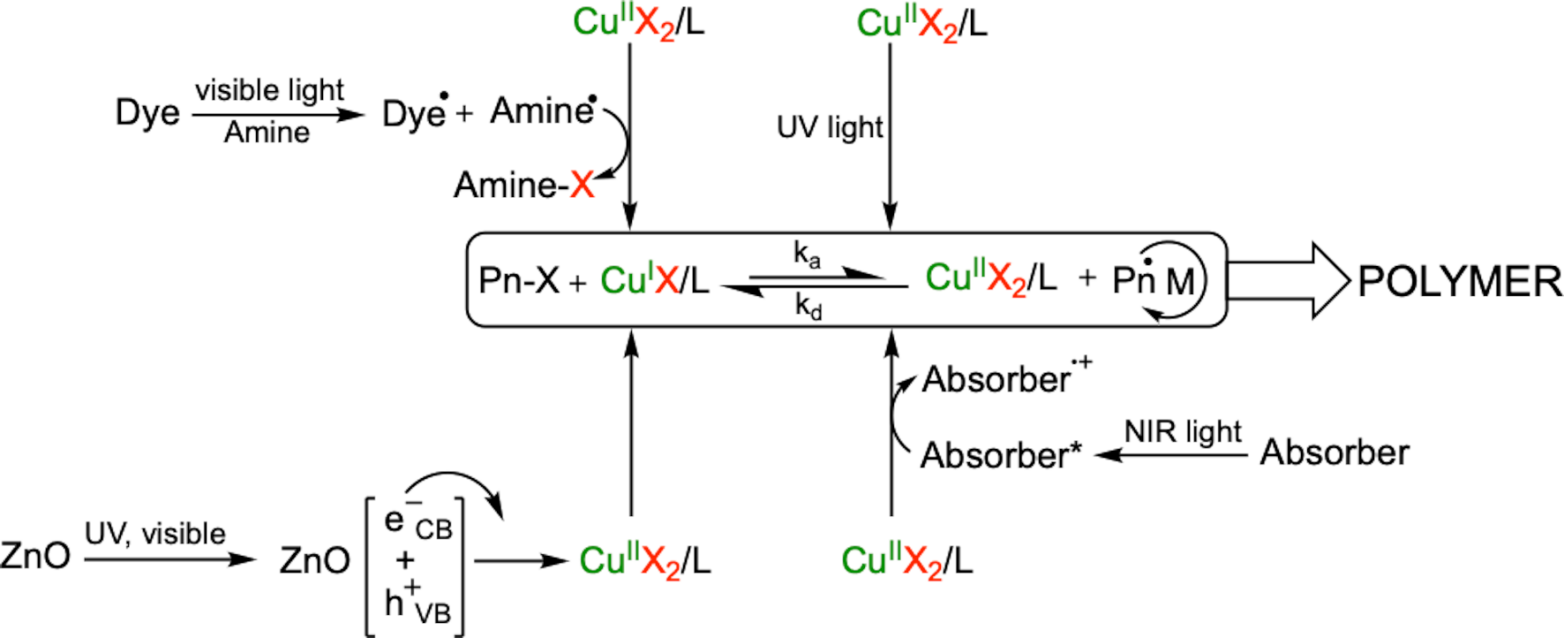
Different modes of photoinitiated ATRP using UV, visible and NIR light.

In addition to those approaches, photo-induced ATRP has been widely used to synthesize well-defined polymers by controlled radical polymerization either with or without heavy metal ions. In this process, basically, in the presence of photoactive materials (**PA**s) such as photoinitiators, photosensitizers or photoredox catalysts, the photoexcitation of a photoredox system results in formation of reactive radicals. Those reactive radicals add monomer and the polymerization proceeds. Several UV and visible light sensitive compounds in conjunction with or without Cu(II) complexes were used to initiate and control the photo-ATRP process (Scheme 9) [130–137]. In the absence of these photoactive materials, the direct irradiation of Cu(II) was presented for in situ generation of Cu(I) to initiate the polymerization with the alkyl bromide. In this system, the Cu(II) complex was exposed to UV light to form Cu(I) which can react with alkyl halide resulting in generation of reactive radicals and Cu(II), Scheme 9. Those reactive radicals add monomer and deactivated by reaction with Cu(II) resulting in formation of dormant species and Cu(I). Altogether, these reactions must be seen as equilibrium coexisting together.

Researchers also spent much time to develop photo-ATRP procedures working without any metal catalyst; that is the photoinduced metal-free ATRP strategy [132–137]. Latter reference provided a comprehensive overview demonstrating the function of phenothiazine derivatives, perylene, diaryl dihydrophenazines, anthracene or pyrene. In addition, type II photoinitiators including benzophenone, and thioxanthones were shown to realize photoinitiated controlled/living radical polymerization of various monomers in metal free conditions. In the visible region, several dyes (fluorescein, eosin Y, erythrosin B) worked well to mediate ATRP in the presence of amines [72]. The reversibility of the electron transfer steps provides the living nature of the process as well as control over the control of functionality as confirmed by the spectroscopic analyses, and chain extension and block copolymerization experiments [138].

**Scheme 10:**
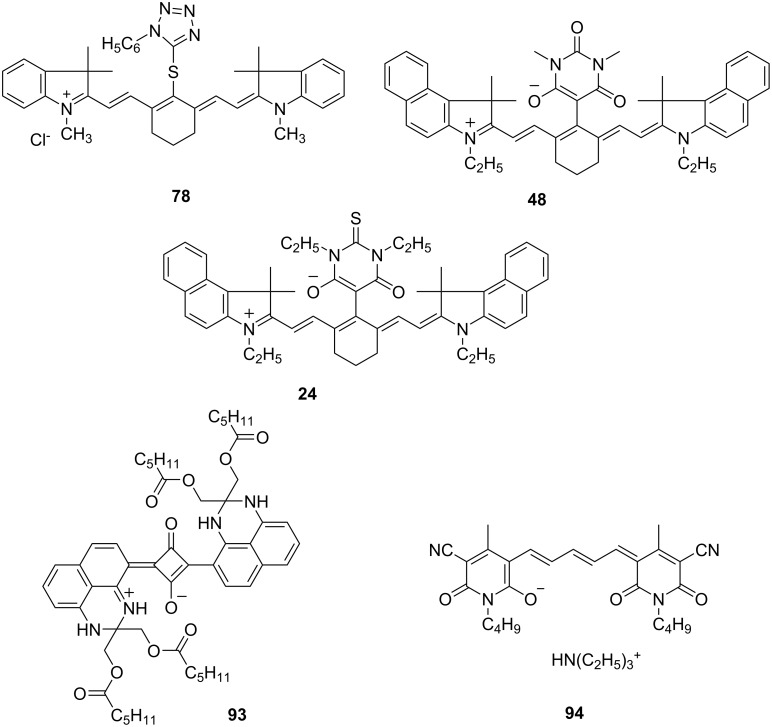
The structure of **Sens** used in photo-ATRP.

The same strategies were also performed in NIR region using NIR sensitizers comprising cationic, zwitterionic and anionic patterns shown in Scheme 10. The polymers obtained exhibited quite high molecular weights (more than 2 × 10^5^ g·mol^−1^) and failed to exhibit living character [81]. However, this system exhibited a successful route with Cu(II) catalysts at the ppm scale [81]. Quite recently, NIR light-induced ATRP has been performed at low catalyst concentrations using NIR sensitizers (**Sens**) as photo-reducing agents with NIR LEDs [81]. Among the NIR sensitizers investigated with cationic, zwitterionic, or anionic structures, only the zwitterionic structure **43** comprising a barbiturate moiety exhibited the successful activity under NIR light resulting in formation of polymers with controlled molecular weight characteristics and functionalities. Scheme 10 depicts the respective structural patterns.

**Figure 4:**
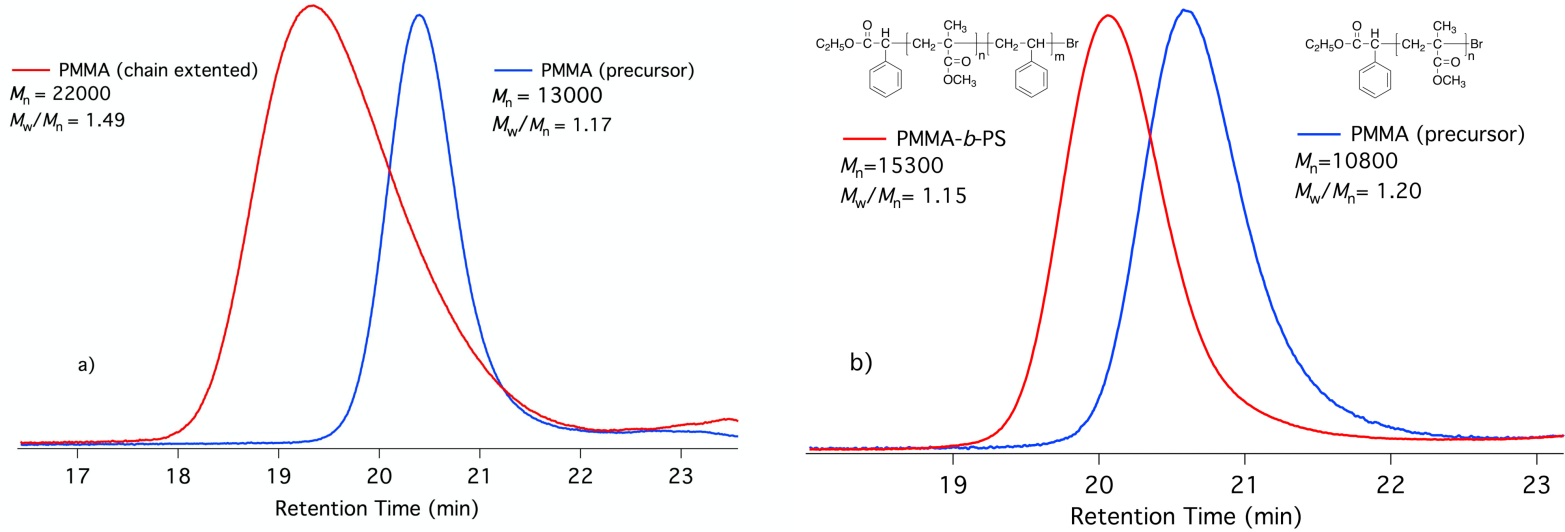
Comparison of the GPC traces of precursor PMMA with a) chain extended PMMA and b) PMMA-b-PS. Conditions for MMA macroinitiator).

UV–vis–NIR spectrum of the reaction components showed strong absorption of **Sens** in the NIR as shown by the huge extinction coefficients in Table 2 vide supra. Additionally, polymerization was not successful in the absence of **Sens** showing the necessity to polymerize NIR-absorber upon exposure with NIR light. This system facilitated control over molecular weight with low dispersity exhibition. Furthermore, chain extension and block copolymerization experiments confirmed the chain end fidelity and therefore the living character of polymerization (Table 3, Figure 4). On/Off experiments demonstrated the light dependency of polymerization [81].

**Table 3:** NIR sensitized photo-ATRP of MMA under different experimental conditions (L: tris(2-pyridylmethyl)amine) with different sensitizers (**Sens**) [81].

**Sens**	[MMA]/[EBPA]/[CuBr_2_]/[L]/[**Sens**]	Cu(II) (ppm)	time (h)	conversion (%)	*M*_n_ (g·mol^−1^)	*M*_w_/*M*_n_

**48**	300/1/0.15/1.65/1.5	500	2	27.2	14000	1.47
**48**	300/1/0.03/0.135/0.3	100	24	44.3	13000	1.17
**48**	300/0/0.03/0.135/0.3	100	24	–	–	–
**48**	300/1/0.03/0.135/0	100	24	–	–	–
**48**	300/1/0/0/0.3	100	24	26.7	400000	2.47
**78**	300/1/0.03/0.135/0.3	100	24	–	–	–
**24**	300/1/0.03/0.135/0.3	100	24	–	–	–
**93**	300/1/0.03/0.135/0.3	100	24	–	–	–
**94**	300/1/0.03/0.135/0.3	100	24	–	–	–

**Figure 5:**
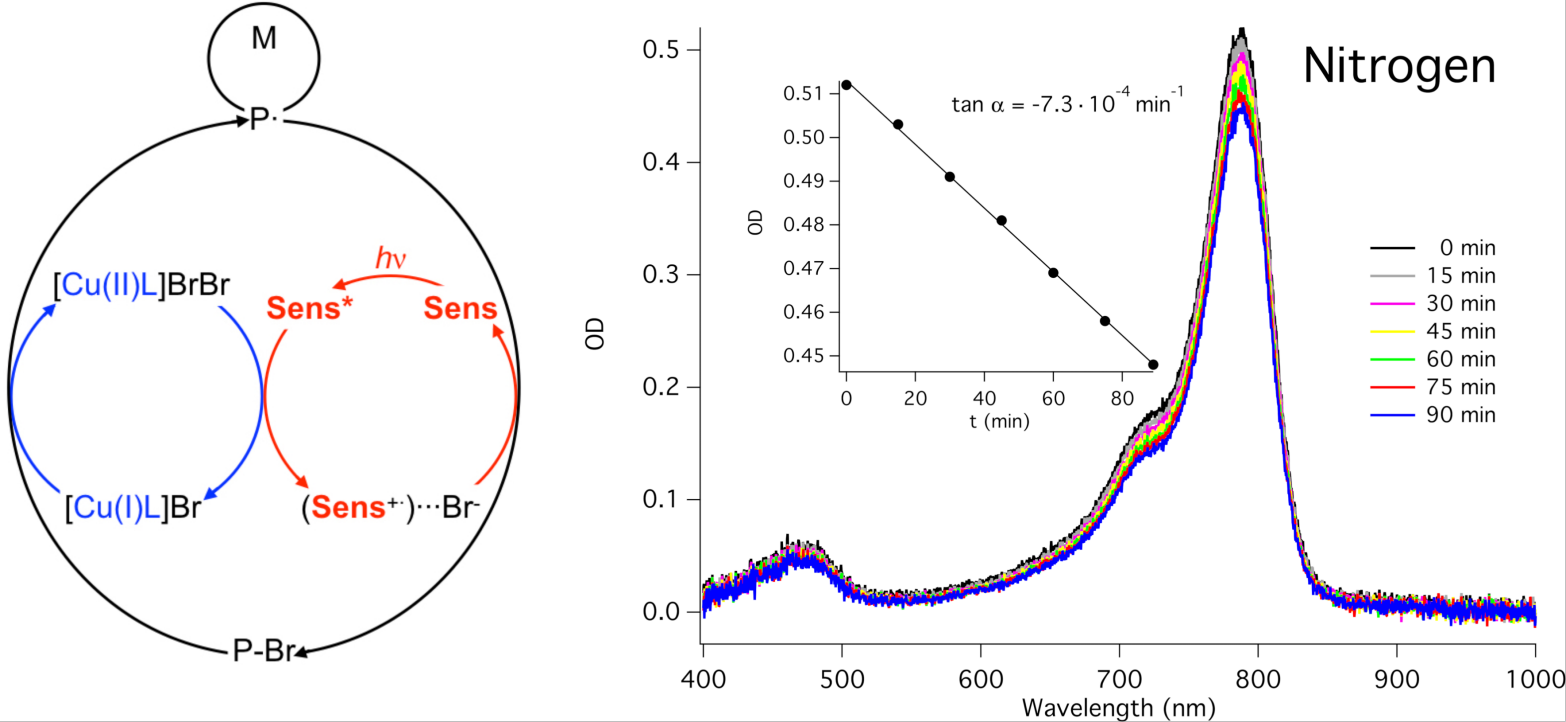
Spectral changes of the solution of **48** in the presence of [Cu(L)]Br2 (L: tris(2-pyridylmethyl)amine) and ethyl α-bromophenylacetate (EBPA) in DMF upon irradiation at 790 nm under nitrogen).

The proposed mechanism (Figure 5) involves the excited state of the sensitizer which directly reduces Cu(II) to Cu(I). The combination of Cu(I) and R–X continues as usual in ATRP generating reactive radicals and halide anion [81]. These radicals add monomer resulting in formation of polymer radicals. The electron transfer from the formed halide anion to the cation radical **Sens**^+•^ forms **Sens** back in the cycle. The system exhibited photocatalytic behavior with no big changes of absorption for 90 min under aerobic and anaerobic conditions. However, no polymerization occurred under air showing the inhibition of polymerization by oxygen [81].

This described system opens the possibility to synthesize well-defined polymeric materials in a green way due to the photochemical stability, low copper-catalyst concentration and the use of NIR light. Taking advantage of good penetration of NIR light, this approach provides also the opportunity to embed UV absorbing materials in coatings and new pathways for bio-applications. Remarkable has been still the fact that this kind of photo-ATRP only worked with **48** carrying a barbiturate group. Such moieties interact with Cu(II) resulting in the formation of complexes as reported by Zwikker already in the 1930’s [139]. This special interaction occurring in particular between **Sens 48** and Cu(II) helps to bring the reactants together and may explain the high selectivity in the NIR-sensitized photo-ATRP of this system. Again, the other sensitizers studied namely **78**, **24**, **93** and **94** showed no activity.

#### NIR light sensitized copper catalyzed azide–alkyne click reactions

**Scheme 11:**
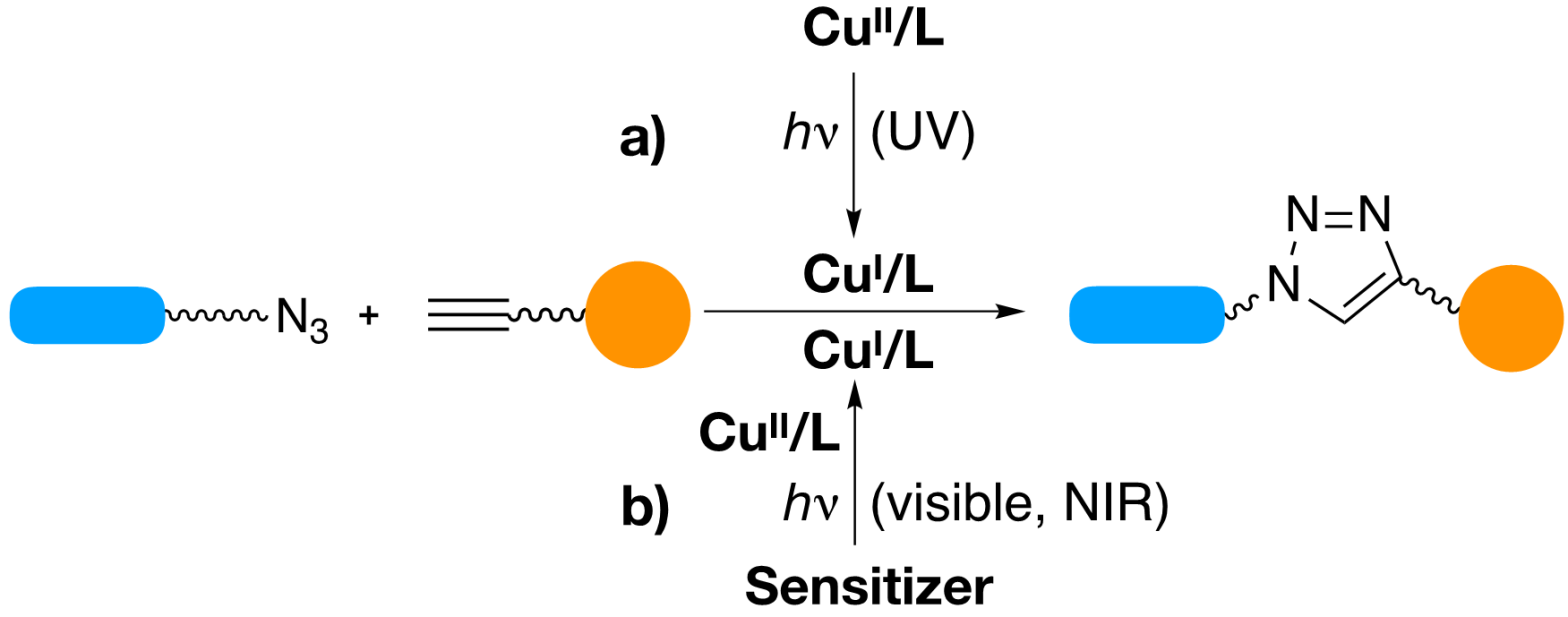
Photoinduced CuAAC reactions in which photochemical reactions result in formation of the Cu(I) catalyst. UV exposure photolysis of Cu(II) results in direct formation of the catalyst for the click reaction while photolysis with visible or NIR light requires the use of a sensitizer resulting in reduction of Cu(II) to Cu(I).

In 2001, the 1,3-dipolar cycloaddition reaction of organic azides and alkynes has gained considerable attention in the fact they are simple to perform and work well under many conditions with high yields [140–142]. The different applications of Cu(I) catalyzed click chemistry have previously reviewed [142–145] while the general system was introduced more than 15 years ago [140–141]. Additional work was performed to develop methods for photoinduced generation of Cu(I) using Cu(II) without any sensitizer or in combination with different sensitizers for this type of click chemistry [143–146]. This mainly worked with either UV or visible light and helped to overcome the requirement of Cu(I) catalyst as substrate. Scheme 11 summarizes the reaction mechanism that includes NIR exposure as well [147] .

NIR sensitizers can also participate in photo-induced mediated click chemistry based on Cu(II), see Scheme 11. The successful electron transfer reaction from the excited NIR sensitizer to the Cu(II) complex for photoinduced ATRP [81] brought the idea to use NIR sensitizers as photoreducing agents for the generation of Cu(I) complex. We applied this system for NIR-light-induced copper–azine–alkyne (CuAAC) reactions for the synthesis of low molar-mass organic compounds and polymeric materials [147]. Different cyanines comprising either neutral or cationic structures were used to activate CuAAC reaction under NIR light (Scheme 11). Again, the sensitizer comprising a cyanine with a barbiturate group (**2**) showed the best activity for the formation of Cu(I) by photoreduction of Cu(II) and worked for the synthesis of low molecular weight compounds and polymers with high yields [147].

**Scheme 12:**
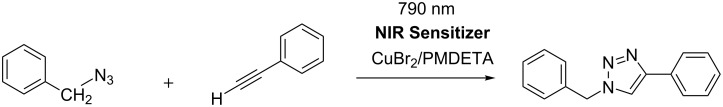
Model reaction between benzyl azide and phenyacetylene using the absorber **48** as NIR sensitizer at 790 nm.

Initially, the successful reaction was observed with benzyl azide and phenylacetylene as model compounds. The mixture including NIR sensitizers together with CuBr_2_/PMDETA, benzyl azide and phenylacetylene were irradiated at 790 nm under anaerobic conditions for 2 hours (Scheme 12). Expectedly, the reaction using **1** as sensitizer failed to show the activity in this system. On the other hand, using **2** as photosensitizer in conjunction with Cu(II) complex showed successful reactivity with almost complete conversion (99%) confirmed by ^1^H NMR.

**Figure 6:**
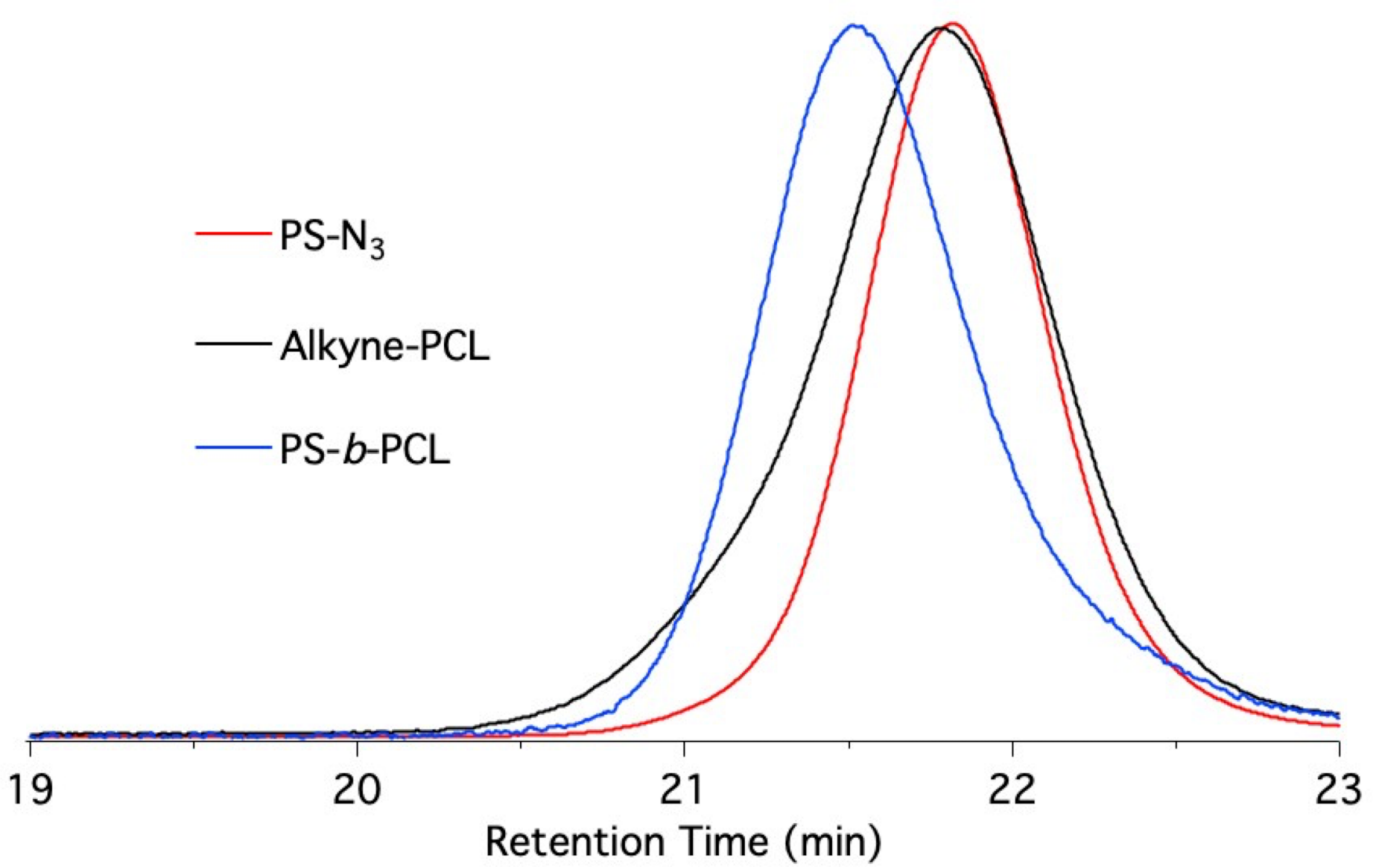
Block copolymerization of the precursors **PS-N3** and **Alkyne-PCL** results in the block copolymer **PS-*b*-PCL** as shown by the respective GPC curves.

Extension of this model reaction to polymers opens the possibility for tailor-made polymer synthesis by click chemistry resulting in formation of block copolymers. This type of copolymer synthesis was performed by reacting polystyrene-azide (**PS-N3**) with alkyne-terminated poly(ε-caprolactone) (**Alkyne-PCL**) in the presence of the barbiturate sensitizer **48** and CuBr_2_/PMDETA. The structure of block copolymer was confirmed by ^1^H NMR analysis and GPC chromatograms of precursors and the resulting block copolymer displayed a unimodal distribution indicating the absence of any other reactions during the click process. The shift of the molecular weight region also showed the successful photoinduced blocking process (Figure 6). This reaction facilitated to connect a hydrophilic and a hydrophobic block by the disclosed click chemistry.

**Figure 7:**
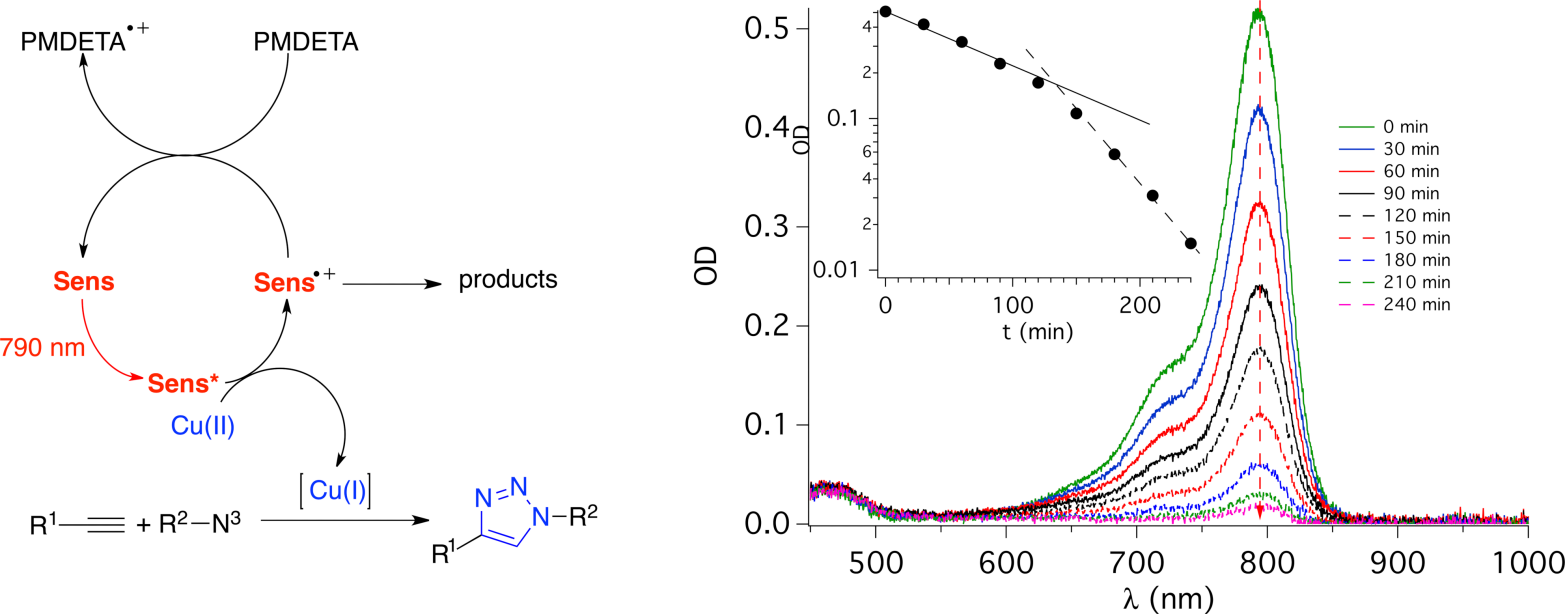
UV–vis–NIR absorption changes of the solution of **48** in the presence of PMDETA, phenylacetylene and benzyl azide in DMSO upon exposure at 790 nm (under nitrogen).

Figure 7 proposes the mechanism based on experimental and spectroscopic investigations. The photoinduced electron transfer from the excited state of the absorber reduces the Cu(II) complex resulting in Cu(I). This catalyzes the CuAAC reaction showing successful formation of the triazole moiety [147]. Photolysis of a mixture comprising benzyl azide, phenylacetylene, and Cu(II)Br_2_/PMDETA and **43** was also studied in a photobleaching experiment. Though the absorption decreases, its lowering is relatively small in this time frame since the reaction with **43** and an iodonium cation resulted in a disappearance of the absorption within less than a minute using the same light source. This smaller decrease of the absorption maximum indicated the capability of the formed cation radical either to decompose in side reactions.

In this work, the use of NIR sensitizer in the photo-CuAAC reaction was shown in the near infrared region from model organic compounds to various macromolecular reactions including polymer end-group functionalization and block copolymer formation with the use of appropriate azide and alkyne click components. This system can be easily implemented in biological and synthetic materials particularly when surface patterning and incorporation of specific groups are required as NIR light is used to induce the click reaction.

This photoreaction possesses also potential to build up polymer networks with almost unique length of the distance between junctions. Such materials should exhibit less brittleness compared to those obtained in a process where chain propagation and termination control polymer formation; that is free radical polymerization. First attempts were made to synthesize such networks applying UV radiation, but this still required some heat treatment after exposure to finalize network formation. The systems disclosed in this contribution also offer enough heat provided in the non-radiative deactivation process, which is higher than ≈80%, Table 2. In addition, it also facilitates embedding of fillers covering an absorption in the UV.

### Chemistry 4.0

#### General remarks [67]

**Scheme 13:**
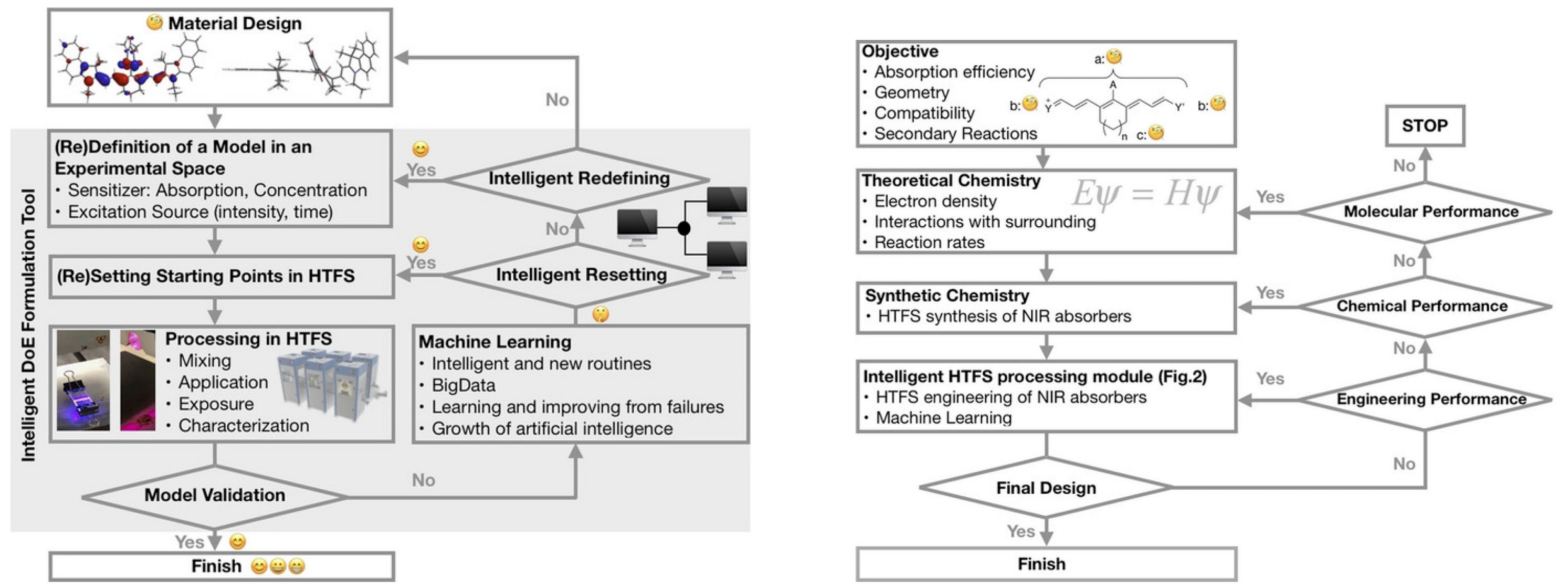
Workflow to design and process new materials in a setup based on an intelligent DoE to develop technologies. Combination of chemistry (theoretical and synthetic chemistry), engineering and informatics creating artificial intelligence results in intelligent tools for development of new materials. NIR technology may benefit from these developments.

Chemistry 4.0 bases on the idea of Industry 4.0 as introduced by the German association VCI (“Verband der Chemischen Industrie e.V.”). It discloses the industrial development starting with Chemistry 1.0 [148] in the 18^th^ century where fossil energy (coal) and chemical processes (firing) generated mechanical power for industrial manufacturing. This is known as founder’s time or “Gründerzeit” relying on coal chemistry. Together with petrochemistry, Chemistry 2.0 was introduced for scale up of production and introduction of new classes of materials such as polymers. Modern development of the industrial society started production of fine chemicals resulting in opening of new ways of industrial chemistry in the early 1980s, which is known as Chemistry 3.0. The development of the internet and new powerful hard- and software where also mobile devices can compete in some applications with desktop computers have moved the focus to develop digital-based production technologies; that is Chemistry 4.0. This digital revolution of the chemical industry stands for sustainable recycling or sometimes called circular economy where waste serves as renewable source or feedstock of new production cycles. Such rapid developments will help to double the production volume as forecasted by 2040. Digitalization will contribute to get these goals. It also uses artificial intelligence (A.I.); that is software helping to learn from huge data sets. Machine learning (ML) represents a sub-group in these tools available. It also helps bring the classical design of experiment (DoE) into a next step which is named intelligent design of experiment (iDoE). NIR absorbers may uptake a function in these workflows since they have been used in coating formulations to design light sensitive materials used in CtP [10–14,66]. Scheme 13 depicts a typical setup of an iDoE [67].

Absorber parameters based on concentration, absorption, and geometry importantly influence the workflow while the radiation source such as an LED or a laser provide the necessary excitation wavelengths and radiation intensity. High Throughput Formulation screening (HTFS), which still relates to automation and therefore Industry 3.0 standards, helps to process them in coating formulations where engineering parameters such as mixing, stirring, and characterization add as more variables to define parameter settings. The measured data train a defined digital model, which correlates input factors and characterization answers. Data analysis of this large data set occurs in a relatively short time frame where each experiment contributes to the model resulting in improvement. A classical DoE ends at this step resulting in information about the success of this experiment. However, an intelligent DoE applies advanced algorithms and improves by several iteration steps the model while its uncertainty becomes smaller. Such routines can predict results in smaller confidence intervals while the model improves in each iteration step to give the definition of new formulations. In other words, the program learns from failures where artificial intelligence and big data sciences uptake a major function. Such routines tolerate variations where robust formulations were applied in production cycles [66].

NIR sensitizers can be also found in Industry 4.0 applications, which relates to Chemistry 4.0 with focus on digitalization of chemical processes. It was translated from the more general term Industry 4.0. The objective is the acceleration and automization of activities like research and development of new materials, quality control, manufacturing processes, service for the customer and so on by devices setting up communication network called internet of things. Acquisition and sharing of data in its digital form for the data analysis is the key task introducing Chemistry 4.0 concepts. Including the characteristics of the materials such as photophysical and photochemical data of the cyanine based NIR absorbers can be seen as one illustrative example, which shows the benefits from the analysis of these data explaining the influence of the large numbers of factors of the absorber material on the processes being described in the preceding chapters. This considers small changes on the molecular pattern resulting in evidence alteration of material characteristics like deactivation processes, solubility, electrochemical properties, etc. and parameters in the area of application [67].

### Digitalization, machine learning and Industry 4.0

Data analysis is the key aspect in the digitalization in chemistry since it derives models from the real existing chemical system. If the data cloud cover the entire area of interest and all influencing parameters, the model, also called digital twin, is capable to predict characteristics of a material or a process based on the experience from real experiments. The far field of machine learning algorithm has already been expanded to application areas like theoretical chemistry and organic synthesis to train models on data sets derived from experimental data and common rules in computational chemistry [149–151]. It has been shown that these models can compete with calculations from chemical laws and increment methods [151–153].

**Scheme 14:**
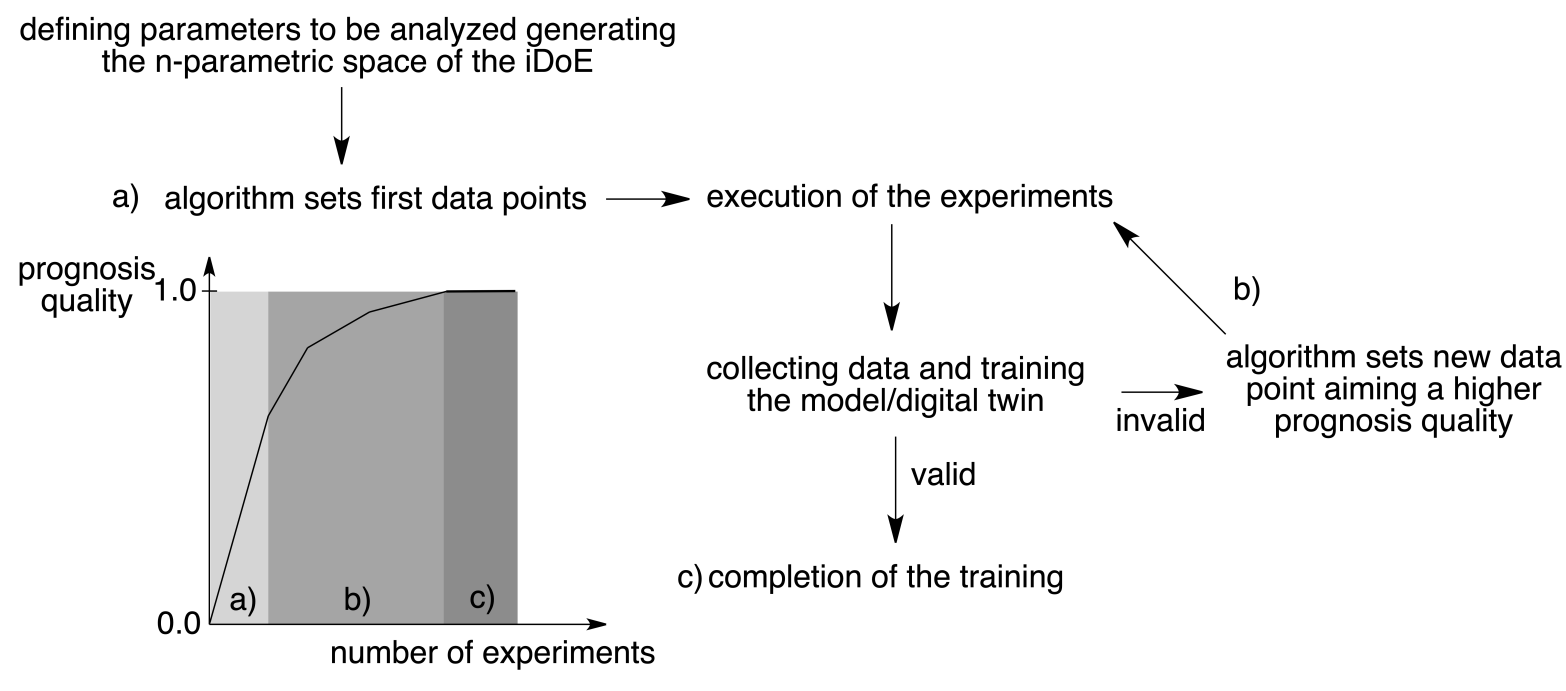
Illustration of the iDoE setting up experiments suggested and analyzed by the A.I. After defining the space of the iDoE by the scientist the algorithm will suggest the starting points (a) and check the validity of the model. In case of a low prognosis quality the A.I. set new data points in experimental spaces with a lack of information (b) improving the model. The task will be finished if the model is capable to predict the outputs based on the inputs within a desired boundary (c).

Further development of algorithms led to innovations, which are useful for the design-of-experiment (DoE) [154] rather than typically known factorial DoE. First reports claim a faster converging of the prognosis quality due to an intelligent design of experiments (iDoE) within the chosen boundaries even if a high number of experimental factors are included [155]. Scheme 14 shows a schematic diagram of the iDoE planning experiments as each adaption with a smaller number of experiments proposed by the A.I. Usually the sampling of the experiments in a DoE is done entirely before execution of the experiments based on the chosen model and chemical system to be analyzed. In contrast, the sampling of experiments during the execution of experiments and training of the model in an adaptive way offers a development of the iDoE based on the history of experiments being already executed. The parameters of the input will be set in spaces of the iDoE with a high uncertainty increasing the prognosis quality. In this direction, a predictive model can be achieved with a number of experiments as low as possible. The execution and training have to be aborted if the prognosis quality does not further increase. At best, this gives a valid model with a prognosis quality close to unity. Based on accurate data, a digital twin can be created for predicting characteristics varied by the input parameters also giving a confidence interval of the prediction.

**Scheme 15:**
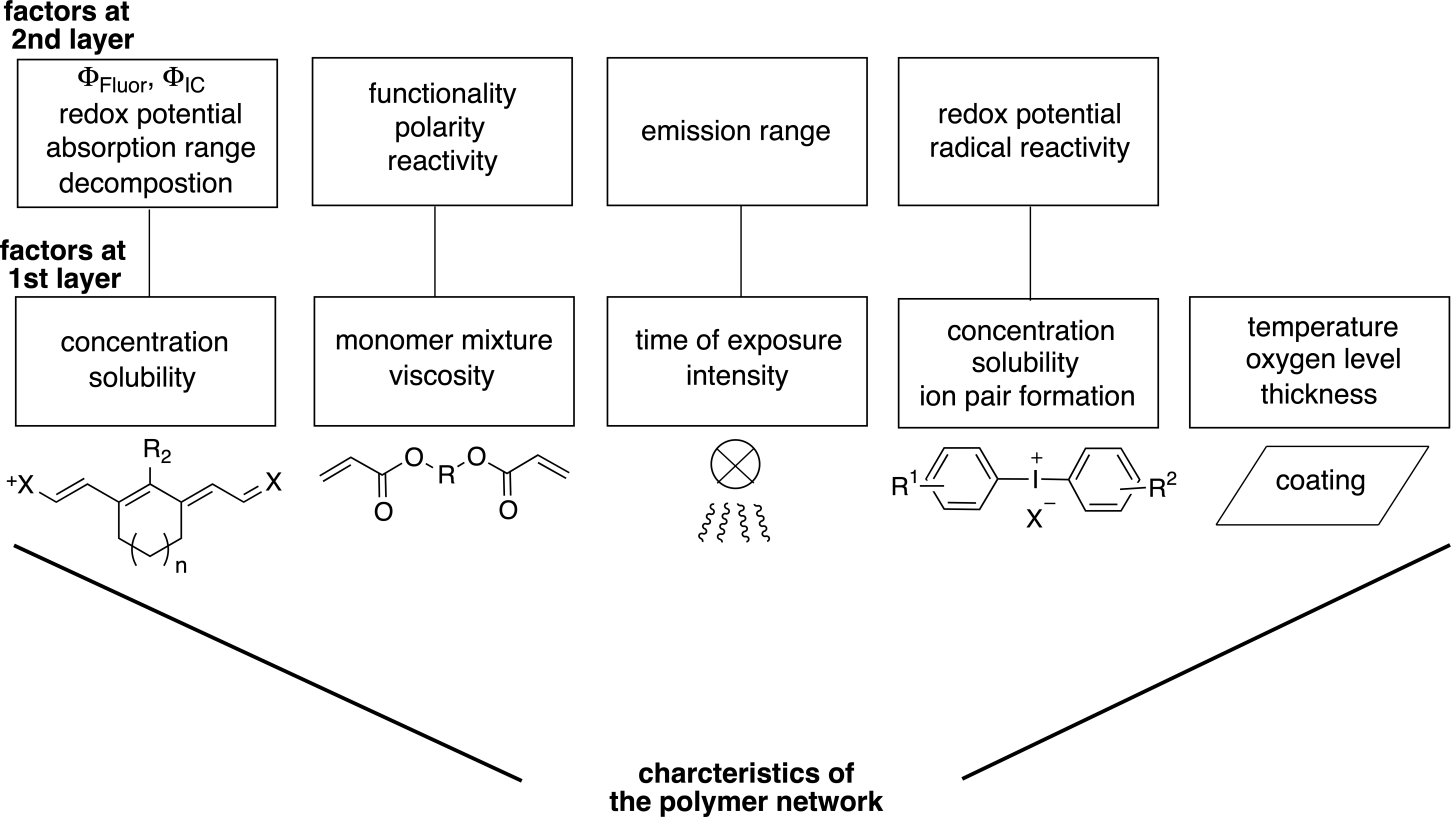
Classification of the factors for the formation of polymer networks by NIR-photocuring depending on the absorber material, the monomers, parameters of the light source, the coinitiator and surrounding conditions. Each position consists of factors directly influencing the process and properties of the material chosen for the formulation at the 2nd layer. A digital twin created by data analysis using machine learning algorithms can describe the relation of the factors in sum.

In engineering processes, like photonic or thermal activation in a photoreactor or applied as coating films, the final properties are influenced by material properties and process parameter. Both types of influences can be introduced into one digital twin and analyzed by the iDoE. In case of applications using heptamethine cyanine absorbers inducing physical processes [16–17,54,58,64] or chemical reactions [5,81,147] the properties of the absorber are important factors besides other coming from distinct ingredients [5–6,10–14,54,63–65]. On the other hand, the analytical solution fails by generating a robust model because such complex relations cannot be described by one mathematical expression available from laws. However, a valid mathematical expression, with Y as the characteristic of the product, according to Equation 9 can be acquired by the empirical method applying machine learning algorithms. For this reason, a graphical description in Scheme 15 takes up again the complexity of the photopolymerization by photoelectron transfer reactions using near infrared LEDs as described vide supra.

[9] $$\begin{array}{c} Y=\sum_{i=1}^{n}f_{i}\left(x_{\text{process}\text{,i}}\right)+\sum_{j=1}^{m}f_{j}\left(x_{\text{material}\;\text{property}\text{,j}}\right)\\ +\sum_{k=1}^{l}f_{k}\left(x_{\text{concentration}\text{,k}}\right) \end{array}$$

*x*_process,i_ = process parameters; *x*_material property,j_ = parameters from material properties of the ingredients/substrates; *x*_concentration,k_ = concentration parameters from the ingredients/substrates

The factors can be divided into certain sub-layers to influence the desired polymer properties. The formulation (e.g., concentrations) and the processing parameters (e.g., intensity, temperature) influence these results directly. However, properties of the material being chosen are factors in the second layer and have an impact on the predictability of the digital twin, too. Every detail can be explained by the experimental findings for each factor but is limited at forecasting the sum of these interacting factors.

On the other side the digital twin from this chemical operation derived by the machine learning algorithm will adaptively increase the prognosis quality if data of each parameter have been integrated into the iDoE. Also, the interactions of factors like the overlap of emission range of the light source and absorption range of the cyanine are taken into account. Again, NIR absorbers play a crucial role in these complex flow charts. Their material properties such as absorption wavelength, absorption coefficient, emission characteristics and electrochemical data enable them in large workflows used for ML.

## Conclusion

This overview clearly demonstrates the potential of NIR absorbers for distinct applications although NIR has been still in its infancy in the field of photopolymerization. A certain existing lack regarding the function of light furthermore confirms such opinions since NIR light has been always considered as source to provide heat and not to initiate chemical and physical processes of molecules. This has surprised since several applications were almost reported until 2013 [57] after the first commercialization of CtP-technology applying NIR-lasers [11,54]. It will hopefully not need such a long period to expand the applicability of NIR absorbers/sensitizers for different purposes.

The compiled photophysical data in this overview will be a helpful toolbox for future design of NIR absorbers; that is absorption and emission maxima, extinction coefficient, and emission quantum yield. Electrochemical data complement the pattern. In addition, the compatibility of the surrounding matrix possesses a major function to transfer NIR absorbers for different applications. This has been often not considered with necessary care. From this point of view, organic chemistry possesses a key to tailor made the properties regarding absorption and solubility as shown by the examples in this overview. Since cyanines exhibit a positive charge in the methine chain, there exist huge possibilities to provide NIR absorbers whose solubility can nicely fit for distinct applications just be change of the anion. This can exhibit either more hydrophobic or hydrophilic properties. Modern tools based on machine learning and A.I. will help to accelerate the development of this field in a shorter time frame. Thus, prediction of molecule properties by quantum chemical methods, organic synthesis, the combination of photophysical data and the functions of NIR absorbers in several applications favors digitalization of chemistry based on the aforementioned described possibilities of machine learning.

Further progress will appear regarding additional uses in industrial applications. Though such materials have been almost available at large scale, more synthetic efforts based on ML in combination with theoretical tools to design molecules will drive this field towards more practical use.

## Supporting Information

File 1 Information of the electrochemical measurements and the determination of photophysical data.

## References

[1] Mustroph H (2014). Dyes, General Survey. Ullmann's Encyclopedia of Industrial Chemistry.

[2] Hamer F E (1964). The Cyanine Dyes and Related Compounds.

[3] Fabian J, Nakazumi H, Matsuoka M (1992). Chem Rev.

[4] Sturmer D M, Taylor E C (1977). Syntheses and Properties of Cyanine and Related Dyes. Chemistry of Heterocyclic Compounds.

[5] Schmitz C, Halbhuber A, Keil D, Strehmel B (2016). Prog Org Coat.

[6] Kocaarslan A, Kütahya C, Keil D, Yagci Y, Strehmel B (2019). ChemPhotoChem.

[7] James N S, Chen Y, Joshi P, Ohulchanskyy T Y, Ethirajan M, Henary M, Strekowski L, Pandey R K (2013). Theranostics.

[8] Choi H S, Nasr K, Alyabyev S, Feith D, Lee J H, Kim S H, Ashitate Y, Hyun H, Patonay G, Strekowski L (2011). Angew Chem, Int Ed.

[9] Lipowska M, Patonay G, Strekowski L (1993). Synth Commun.

[10] Vollmann H W (1980). Angew Chem.

[11] Baumann H, Hoffmann-Walbeck T, Wenning W, Lehmann H-J, Simpson C D, Mustroph H, Stebani U, Telser T, Weichmann A, Studenroth R (2015). Imaging Technology, 3. Imaging in Graphic Arts. Ullmann's Encyclopedia of Industrial Chemistry.

[12] Baumann H (2015). Chem Unserer Zeit.

[13] Strehmel B, Ernst S, Reiner K, Keil D, Lindauer H, Baumann H (2014). Z Phys Chem.

[14] Brömme T, Schmitz C, Oprych D, Wenda A, Strehmel V, Grabolle M, Resch-Genger U, Ernst S, Reiner K, Keil D (2016). Chem Eng Technol.

[15] Strehmel B, Schmitz C, Bromme T, Halbhuber A, Oprych D, Gutmann J S (2016). J Photopolym Sci Technol.

[16] Schmitz C, Strehmel B (2017). ChemPhotoChem.

[17] Schmitz C, Gökce B, Jakobi J, Barcikowski S, Strehmel B (2016). ChemistrySelect.

[18] Pitz H (2003). Vorrichtung und Verfahren zur Zuführung von Strahlungsenergie auf einen Bedruckstoff in einer Flachdruckmaschine.

[19] Schlörholz M, Keil D, Pitz H (2008). Verfahren zum Trocknen von Druckfarbe auf einem Bedruckstoff.

[20] Ernst S, Peiter G D, Pitz H, Reiner K, Mistol J, Schlörholz M (2007). IR dyes and laser markable articles comprising such IR dyes.

[21] Nakhaei M R, Mostafa Arab N B, Kordestani F (2012). Adv Mater Res.

[22] Brunnecker F, Sieben M (2010). Laser Tech J.

[23] Wissemborski R, Klein R (2010). Laser Tech J.

[24] Jones I A, Taylor N S, Sallvanti R, Griffith J (2014). Int J Res Eng Technol.

[25] Ernst S, Keil D, Reiner K, Senns B (2018). NIR-Absorber Additive für das Laserstrahlschweißen von Kunststoffen.

[26] Pérez-Barrado E, Darton R J, Guhl D (2018). MRS Commun.

[27] Patel C, Patel A J, Patel R C (2017). Int J Sci Res Dev.

[28] Loccufier J (2018). Developing Agent Precursor for Laser Markable Composiitons.

[29] Waumans B, Geuens I, Callant P, Van Aert H (2015). Security documents and colour laser marking methods for securing them.

[30] Thaker J (2011). Laser fusible coating compositions used for laser marking systems and methods.

[31] Koenemann M, Boehm A, Pschirer N G, Qu J, Mattern G (2007). Substituierte Rylenderivate.

[32] Brooker L G S, Mees C E K, James T H (1966). Sensitizing and Desensitizing Dyes. The Theory of the Photographic Process.

[33] Sturmer D M, Heseltine D W, James T H (1977). Sensitizing and Desensitizing Dyes. The Theory of the Photographic Process.

[34] Keller K, Kampfer H, Matejec R, Lapp O, Krafft W, Frenken H, Lührig H, Scheerer R, Heilmann M, Meckl H (2000). Photography. Ullmann's Encyclopedia of Industrial Chemistry.

[35] Ficken G E, Venkataraman K (1971). Cyanine Dyes. The Chemistry of Synthetic Dyes.

[36] Berlin L, Riester O (1972). Methoden zur Herstellung von Cyaninen (Polymethinen). Methoden der organischen Chemie.

[37] Sturmer D M, Weissberger A, Taylor E C (1977). Syntheses and Properties of Cyanine and Related Dyes. The Chemistry of Heterocyclic Compounds.

[38] Sturmer D M (1979). Cyanine Dyes. Kirk-Othmer Encyclopedia of Chemical Technology.

[39] Raue R, Riester O (1979). Methinfarbstoffe. Ullmanns Enzyklopädie der Technischen Chemie.

[40] Mishra A, Behera R K, Behera P K, Mishra B K, Behera G B (2000). Chem Rev.

[41] Steiger R, Hediger H, Junod P (1980). Photogr Sci Eng.

[42] Beretta P, Jaboli A (1974). Photogr Sci Eng.

[43] Herz A H (1974). Photogr Sci Eng.

[44] Herz A H (1974). Photogr Sci Eng.

[45] Norland K, Ames A, Taylor T (1970). Photogr Sci Eng.

[46] McRae E G (1961). Aust J Chem.

[47] Kasha M, Rawls H R, Ashraf El-Bayoumi M (1965). Pure Appl Chem.

[48] McRae E G, Kasha M (1958). J Chem Phys.

[49] Hestand N J, Spano F C (2018). Chem Rev.

[50] Scheibe G (1937). Angew Chem.

[51] Jelley E E (1937). Nature.

[52] Dewar M J S (1950). J Chem Soc.

[53] Knott E B (1951). J Chem Soc.

[54] Strehmel B, Brömme T, Schmitz C, Reiner K, Ernst S, Keil D (2015). NIR-Dyes for Photopolymers and Laser Drying in the Graphic Industry. Dyes and Chromophores in Polymer Science.

[R55] 55Color relates to the spectral sensitivity of the human eye covering 400–700 nm. The cones comprise three sensitive pigments for blue, green and red. The combined stimulus signal of the three visual pigments results in perception of color. An equally strong stimulus signal in the green and red areas produces the color sensation yellow if these colors possess the same intensity. The fact that one interprets 770 nm still as red relates to the fact the eye translates this heat related radiation into a slight red.

[56] Yuan Z, Lee S-L, Chen L, Li C, Mali K S, De Feyter S, Müllen K (2013). Chem – Eur J.

[57] Wang Z Y (2013). Near-Infrared Organic Materials and Emerging Applications.

[58] Schmitz C, Strehmel B (2019). J Coat Technol Res.

[59] Lee H, Berezin M Y, Henary M, Strekowski L, Achilefu S (2008). J Photochem Photobiol, A.

[60] Benson R C, Kues H A (1977). J Chem Eng Data.

[61] Pouradier J (1964). J Chim Phys.

[62] Kim J S, Kodagahally R, Strekowski L, Patonay G (2005). Talanta.

[63] Brömme T, Schmitz C, Moszner N, Burtscher P, Strehmel N, Strehmel B (2016). ChemistrySelect.

[64] Schmitz C, Oprych D, Kutahya C, Strehmel B, Lalevée J, Fouassier J-P (2018). NIR Light for Initiation of Photopolymerization. Photopolymerisation Initiating Systems.

[65] Schmitz C, Pang Y, Gülz A, Gläser M, Horst J, Jäger M, Strehmel B (2019). Angew Chem, Int Ed.

[66] Plümer L, Korolik P, Koifman D, Baumann H, Strehmel B (2011). Method for automated control of processing parameters.

[67] Strehmel B, Schmitz C, Cremanns K, Göttert J (2019). Chem – Eur J.

[68] Nguyen M T, Locas M A (2006). Thermally reactive near-infrared absorbing acetal copolymers, methods of preparation and methods of use.

[69] Roschger P, Michaelis S, Hassenrueck K, Berneth H, Callant P (1995). Verfahren zur Herstellung von Cyaninfarbstoffen.

[70] Baumann H, Ernst U, Goez M, Griesbeck A, Oelgemöller M, Oppenländer T, Schlörholz M, Strehmel B (2014). Nachr Chem.

[71] Ignatyev N D, Kucheryna A D, Welz-Biermann U D, Willner H P D (2005). FAP-Farbstoffe.

[72] Kavarnos G J, Turro N J (1986). Chem Rev.

[73] Aulin Y V, Liu M, Piotrowiak P (2019). J Phys Chem Lett.

[74] Dlott D D (1990). J Lumin.

[75] Mustroph H (2016). ChemPhysChem.

[76] Mustroph H, Ernst S (2011). Chem Unserer Zeit.

[R77] 77Explorative curing studies of powder coatings as pursued in reference [16] showed temperature increase up to 200 °C while no pigment was embedded. Addition of iron oxide pigments significantly resulted in an increase of the temperature during processing up to 350 °C using the same setup.

[78] Lee S, George Thomas R, Ju Moon M, Ju Park H, Park I-K, Lee B-I, Yeon Jeong Y (2017). Sci Rep.

[79] Yan Y, Chen J, Yang Z, Zhang X, Liu Z, Hua J (2018). J Mater Chem B.

[80] Xu Q, Shen Y, Zhang Y, Shao X (2019). Bioorg Med Chem Lett.

[81] Kütahya C, Schmitz C, Strehmel V, Yagci Y, Strehmel B (2018). Angew Chem, Int Ed.

[82] Uo M, Kudo E, Okada A, Soga K, Jogo Y (2009). J Photopolym Sci Technol.

[83] Schwalm R (2007). UV Coatings. Basics, Recents Developments and New Applications.

[84] Romańczyk P P, Kurek S S (2017). Electrochim Acta.

[85] Bonardi A H, Dumur F, Grant T M, Noirbent G, Gigmes D, Lessard B H, Fouassier J-P, Lalevée J (2018). Macromolecules.

[86] Bonardi A-H, Bonardi F, Morlet-Savary F, Dietlin C, Noirbent G, Grant T M, Fouassier J-P, Dumur F, Lessard B H, Gigmes D (2018). Macromolecules.

[87] Bonardi A, Bonardi F, Noirbent G, Dumur F, Dietlin C, Gigmes D, Fouassier J-P, Lalevée J (2019). Polym Chem.

[88] Bonardi A H, Bonardi F, Dumur F, Gigmes D, Fouassier J P, Lalevée J (2019). Macromol Rapid Commun.

[R89] 89In 2013, samples of **34** and **48** were transferred to the lab of Prof. P. R. Ogilby in Åarhus (Denmark) to quantify singlet oxygen formed. He confirmed that there was no singlet oxygen formed. This rules out formation of triplet states that could build singlet oxygen.

[90] Samanta A, Vendrell M, Das R, Chang Y-T (2010). Chem Commun.

[91] Gorka A P, Nani R R, Zhu J, Mackem S, Schnermann M J (2014). J Am Chem Soc.

[92] Odian G (2004). Principles of Polymerization.

[93] Weyts K F, Goethals E J (1988). Polym Bull.

[94] Goethals E J, Van de Velde M, Eckhaut G, Bouquet G (1985). ACS Symp Ser.

[95] Goethals E J, Vlegels M A (1981). Polym Bull.

[96] Goethals E J, Schacht E H, Bruggeman P, Bossaer P (1977). ACS Symp Ser.

[97] Iwai Y, Kunita K (2007). Compound having polymethine-chain structure, image forming material, planographic printing plate precursor, and image forming method using the same, method of making planographic printing plate, and planographic printing method.

[98] Iwai Y, Kunita K (2006). Compound having polymethine-chain structure, image forming material, planographic printing plate precursor, and image forming method using the same, method of making planographic printing plate, and planographic printing method.

[99] Simpson P, Baumann H, Strehmel B (2009). Sensitizer/initiator combination for negative-working thermal-sensitive compositions usable for lithographic plates.

[100] Kropp M A, Baillargeon M, Park K M, Bhamidapaty K, Schuster G B (1991). J Am Chem Soc.

[101] Schuster G B (1990). Pure Appl Chem.

[102] Chatterjee S, Davis P D, Gottschalk P, Kurz M E, Sauerwein B, Yang X, Schuster G B (1990). J Am Chem Soc.

[103] Kabatc J, Zasada M, Paczkowski J (2007). J Polym Sci, Part A: Polym Chem.

[104] Bruder F-K, Fäcke T, Rölle T (2017). Polymers (Basel, Switz).

[105] Bruder F-K, Hagen R, Rölle T, Weiser M-S, Fäcke T (2011). Angew Chem.

[106] Bruder F-K, Deuber F, Facke T, Hagen R, Honel D, Jurberg D, Kogure M, Rolle T, Weiser M-S (2009). J Photopolym Sci Technol.

[107] Sarker A M, Kaneko Y, Neckers D C (1999). J Photochem Photobiol, A.

[108] Kaneko Y, Sarker A M, Neckers D C (1999). Chem Mater.

[109] Sarker A M, Lungu A, Mejiritski A, Kaneko Y, Neckers D C (1998). J Chem Soc, Perkin Trans 2.

[110] Sarker A M, Kaneko Y, Nikolaitchik A V, Neckers D C (1998). J Phys Chem A.

[111] Popielarz R, Sarker A M, Neckers D C (1998). Macromolecules.

[112] Feng K, Zang H, Martin D, Marino T L, Neckers D C (1998). J Polym Sci, Part A: Polym Chem.

[113] Sarker A M, Lungu A, Neckers D C (1996). Macromolecules.

[114] Hassoon S, Sarker A, Polykarpov A Y, Rodgers M A J, Neckers D C (1996). J Phys Chem.

[115] Hauck G, Savariar-Hauck C, Timpe H-J (2000). Kodak Polychrome Graphics.

[116] Berdzinski S, Strehmel B, Strehmel V (2015). Photochem Photobiol Sci.

[117] Strekowski L, Lee H, Mason J C, Say M, Patonay G (2009). J Heterocycl Chem.

[118] Matyjaszewski K, Davis T P (2002). Handbook of Radical Polymerization.

[119] Jiang J, Ye G, Wang Z, Lu Y, Chen J, Matyjaszewski K (2018). Angew Chem, Int Ed.

[120] McKenzie T G, Fu Q, Uchiyama M, Satoh K, Xu J, Boyer C, Kamigaito M, Qiao G G (2016). Adv Sci.

[121] Shanmugam S, Xu J, Boyer C (2016). Angew Chem.

[122] Matyjaszewski K (2012). Macromolecules.

[123] Ribelli T G, Fantin M, Daran J-C, Augustine K F, Poli R, Matyjaszewski K (2018). J Am Chem Soc.

[124] Enciso A E, Fu L, Russell A J, Matyjaszewski K (2018). Angew Chem, Int Ed.

[125] Yang Y, Liu X, Ye G, Zhu S, Wang Z, Huo X, Matyjaszewski K, Lu Y, Chen J (2017). ACS Appl Mater Interfaces.

[126] Matyjaszewski K (2017). Chem Int.

[127] Ribelli T G, Konkolewicz D, Pan X, Matyjaszewski K (2014). Macromolecules.

[128] Ribelli T G, Konkolewicz D, Bernhard S, Matyjaszewski K (2014). J Am Chem Soc.

[129] Dadashi-Silab S, Doran S, Yagci Y (2016). Chem Rev.

[130] Tasdelen M A, Uygun M, Yagci Y (2011). Macromol Rapid Commun.

[131] Tasdelen M A, Uygun M, Yagci Y (2010). Macromol Chem Phys.

[132] Treat N J, Sprafke H, Kramer J W, Clark P G, Barton B E, Read de Alaniz J, Fors B P, Hawker C J (2014). J Am Chem Soc.

[133] Pan X, Fang C, Fantin M, Malhotra N, So W Y, Peteanu L A, Isse A A, Gennaro A, Liu P, Matyjaszewski K (2016). J Am Chem Soc.

[134] Kutahya C, Allushi A, Isci R, Kreutzer J, Ozturk T, Yilmaz G, Yagci Y (2017). Macromolecules.

[135] Aydogan C, Kutahya C, Allushi A, Yilmaz G, Yagci Y (2017). Polym Chem.

[136] Kutahya C, Aykac F S, Yilmaz G, Yagci Y (2016). Polym Chem.

[137] Jockusch S, Yagci Y (2016). Polym Chem.

[138] Stenzel M H, Barner-Kowollik C (2016). Mater Horiz.

[139] Zwikker J L (1933). Pharm Weekbl.

[140] Kolb H C, Finn M G, Sharpless K B (2001). Angew Chem, Int Ed.

[141] Rostovtsev V V, Green L G, Fokin V V, Sharpless K B (2002). Angew Chem, Int Ed.

[142] Wu P, Feldman A K, Nugent A K, Hawker C J, Scheel A, Voit B, Pyun J, Fréchet J M J, Sharpless K B, Fokin V V (2004). Angew Chem, Int Ed.

[143] Tasdelen M A, Yagci Y (2013). Angew Chem, Int Ed.

[144] Gacal B, Akat H, Balta D K, Arsu N, Yagci Y (2008). Macromolecules.

[145] Tasdelen M A, Yilmaz G, Iskin B, Yagci Y (2012). Macromolecules.

[146] Temel G, Aydogan B, Arsu N, Yagci Y (2009). Macromolecules.

[147] Kütahya C, Yagci Y, Strehmel B (2019). ChemPhotoChem.

[R148] 148More about Chemistry 4.0 can be found in “Growth through innovation in a transforming world” published by Verband der Chemischen Industrie VCI, 2017. https://www.vci.de/vci-online/services/publikationen/broschueren-faltblaetter/vci-deloitte-study-chemistry-4-dot-0-short-version.jsp (11 January 2020).

[149] Lelièvre T, Stoltz G (2016). Acta Numerica.

[150] Li L, Baker T E, White S R, Burke K (2016). Phys Rev B.

[151] Grajciar L, Heard C J, Bondarenko A A, Polynski M V, Meeprasert J, Pidko E A, Nachtigall P (2018). Chem Soc Rev.

[152] Rupp M (2015). Int J Quantum Chem.

[153] Grisafi A, Fabrizio A, Meyer B, Wilkins D M, Corminboeuf C, Ceriotti M (2019). ACS Cent Sci.

[154] Cremanns K, Roos D (2017). arXiv.

[155] Cremanns K, Schmitz C, Wagner L (2019). Farbe und Lack.

