# Supplementary material for: Photophysics and photochemistry of NIR absorbers derived from cyanines: key to new technologies based on chemistry 4.0
Source: Beilstein J Org Chem. 2020 Mar 18;16:415–44. doi: 10.3762/bjoc.16.40 (PMC7113544; doi:10.3762/bjoc.16.40)
Supplement: File 1 — Information of the electrochemical measurements and the determination of photophysical data. [file Beilstein_J_Org_Chem-16-415-s001.pdf]

BEILSTEIN JOURNAL OF ORGANIC CHEMISTRY

Supporting Information

for

Photophysics and photochemistry of NIR absorbers derived from cyanines: key to new technologies based on chemistry 4.0

Bernd Strehmel, Christian Schmitz, Ceren Kütahya, Yulian Pang, Anke Drewitz and Heinz Mustroph

Beilstein J. Org. Chem. **2020,** *16,* 415–444. doi:10.3762/bjoc.16.40

Information of the electrochemical measurements and the determination of photophysical data

License and Terms: This is a supporting information file under the terms of the Creative Commons Attribution License (http://creativecommons.org/licenses/by/4.0). Please note that the reuse, redistribution and reproduction in particular requires that the authors and source are credited.
The license is subject to the *Beilstein Journal of Organic Chemistry* terms and conditions: (https://www.beilstein-journals.org/bjoc)

Materials

The NIR-absorbers were available either as catalog products or samples from FEW Chemicals GmbH. Solvents used were purchased as spectroscopic grade purity from Sigma Aldrich.

UV–vis–NIR data

Spectra of the dissolved sensitizers in the monomers were measured with a Cary 5000 from Agilent. Measurements were carried out in 1 × 1 cm quartz cuvettes.

Fluorescence measurements

Fluorescence measurements were pursued in 1 × 1 cm quartz cuvettes. Data were taken with a NIR-fluorescence spectrometer FS920 von Edinburgh Instruments equipped with a Hamamatsu PMT R2658P photomultiplier and double monochromators. A Xe-450W lamp served as excitation source. Sulforhodamine 101 (c = 0.1 M) in HCl/ethanol exhibiting a quantum yield of 1 served as reference. The absorbers were measured with respect to the reference under identical conditions to determine the fluorescence quantum yield. Four samples were prepared for each absorber (OD < 0.1, d = 1 cm)) choosing three distinct excitation wavelengths while the emission of the solvent was subtracted as background. Samples were measured at 22 °C. The fluorescence spectrometer worked in both the emission and excitation side in the correction mode. The fluorescence quantum yield is given in percent data with respect the reference.

Cyclic voltammetry

The oxidation potential of the sensitizers was recorded by cyclic voltammetry (Zennium from Zahner-Elektrik GmbH and a VERSASTAT4-400 from AMETEK served as potentiostats) in acetonitrile ($c_{absorber}$ = 10^{-3} M) with tetrabutylammonium hexafluorophosphate from Sigma Aldrich (0.1 M) as supporting electrolyte against ferrocene as an external standard. The data were taken with a scanning rate of 0.015 $V \cdot s^{-1}$ using platinum disc as a working electrode and Ag/AgCl as reference electrode.
